# Supplementary material for: Efficient urea electrosynthesis from carbon dioxide and nitrate via alternating Cu–W bimetallic C–N coupling sites
Source: Nat Commun. 2023 Jul 26;14:4491. doi: 10.1038/s41467-023-40273-2 (PMC10372083; doi:10.1038/s41467-023-40273-2)
Supplement: Supplementary file 1 — Supplementary Information [file 41467_2023_40273_MOESM1_ESM.pdf]

Supplementary Information

Efficient urea electrosynthesis from carbon dioxide and nitrate via alternating Cu-W bimetallic C–N coupling sites

Yilong Zhao,[1#] Yunxuan Ding,[2#] Wenlong Li,[1,2] Chang Liu,[1] Yingzheng Li,[1] Ziqi Zhao,[1] Yu Shan,[1] Fei Li,[1] Licheng Sun,[1,2,3]* and Fusheng Li[1]*

[1]State Key Laboratory of Fine Chemicals, Institute of Artificial Photosynthesis, DUT-KTH Joint Education and Research Centre on Molecular Devices, Dalian University of Technology, 116024 Dalian, China.

[2]Center of Artificial Photosynthesis for Solar Fuels and Department of Chemistry, School of Science, Westlake University, 310024 Hangzhou, China.

[3]Department of Chemistry, School of Engineering Sciences in Chemistry, Biotechnology and Health, KTH Royal Institute of Technology, 10044 Stockholm, Sweden.

[#]These authors contributed equally: Yilong Zhao, Yunxuan Ding.

*Corresponding authors. Email: sunlicheng@westlake.edu.cn; fusheng@dlut.edu.cn

Table of Contents

Supplementary Methods-- 3

Physical characterisation-- 4

Electrochemical data and analytical methods--- 13

Stability analysis--- 35

Performance comparison-- 47

In situ characterization and mechanism analysis--- 48

Theoretical calculation of DFT--- 55

Supplementary References-- 60

Supplementary Methods

Chemicals. All chemicals and gases were purchased commercially unless otherwise stated. Ar (99.999%), CO_2 (99.999%), CO (99.999%), NO (99.9%), NO_2 (2000 ppm in Ar) and $^{13}CO_2$ (99.5%) were purchased from Dalian Special Gases Co. and Sigma-Aldrich. $Na_2WO_4·2H_2O$ (99.5%), $CuSO_4$ (99.95%), $KHCO_3$ (99.7%), K_2CO_3 (99.99%), KNO_3 (99.0%), KNO_2 (97%), K_2HPO_4 (99%), KH_2PO_4 (99%), EDTA-Na_2 (99%), Na_2CO_3 (99.99%), hydrochloric acid (HCl,37% in H_2O) and hydroxylamine solution (NH_2OH, 50 wt.% in H_2O) were purchased from Aladdin. The reagents used for quantitative analysis include phosphoric acid (H_3PO_4, >85 wt.% in H_2O), sulfuric acid (H_2SO_4, 95%-98%), iron chloride (>99.9%), thiosemicarbazide (99%), sodium hypochlorite solution (active chlorine >10%), salicylic acid (99.5%), sodium citrate dihydrate (99.0%), sodium nitro ferricyanide dihydrate (99.0%), urea (99%), ^{15}N-urea (99%), NH_4Cl (>99.5%), $^{15}NH_4Cl$ (99%), $K^{15}NO_3$ (98%) and diacetylmonoxime (>98%) were produced by Sigma-Aldrich. Urease (~1 U/mg) was gained from Shanghai yuanye Bio-Technology Co., Ltd. Dimethyl sulfoxide (DMSO, ≥99.9%) and absolute ethyl alcohol were supplied by Innochem. All aqueous solutions were prepared with high-purity deionized water (Milli-Q, resistance 18 MΩ cm^{-1}).

Characterization. The morphology and structure of catalysts were investigated by scanning electron microscope (SU8220, HITACHI) and cryogenic transmission electron microscope (Glacios Cryo-TEM 200 kV, Thermo Fisher). The phase structures of the catalysts were obtained by X-ray smart diffractometer (Rigaku SmartLab 9KW) and Renishaw microscopic confocal Raman spectrometer (Thermo Fisher DXR Microscope) using a laser wavelength of 532 nm. Fourier transform infrared (FT-IR) spectra were acquired on a Thermo 6700 infrared spectrometer. The surface composition of the electrode films was investigated using X-ray photoelectron spectroscopy (XPS) on an ESCALAB Xi+ (Thermo Scientific™).

Supplementary Figures

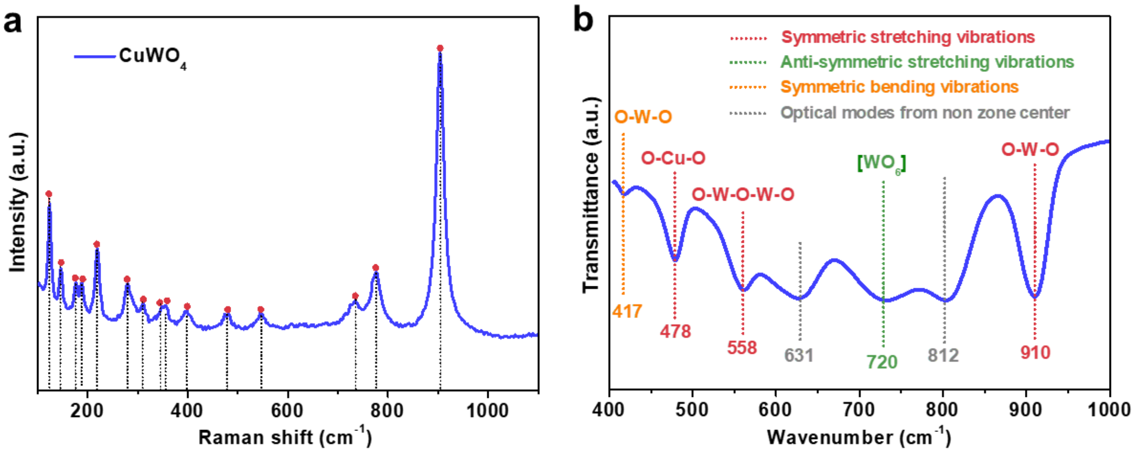

Supplementary Figure 1 The (a) Raman spectrum and (b) FT-IR spectrum of $CuWO_4$ catalyst.

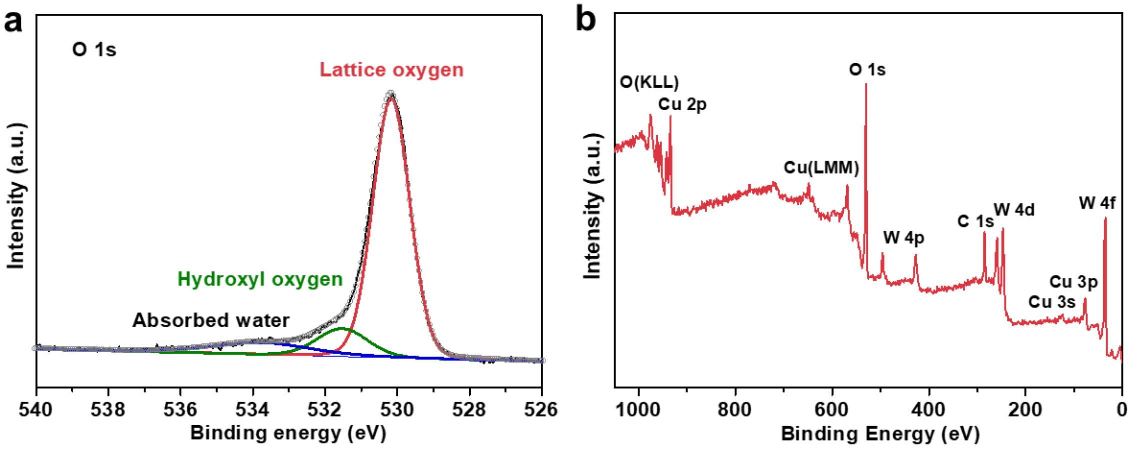

Supplementary Figure 2 XPS (a) O 1s and (b) survey spectra of $CuWO_4$ catalyst.

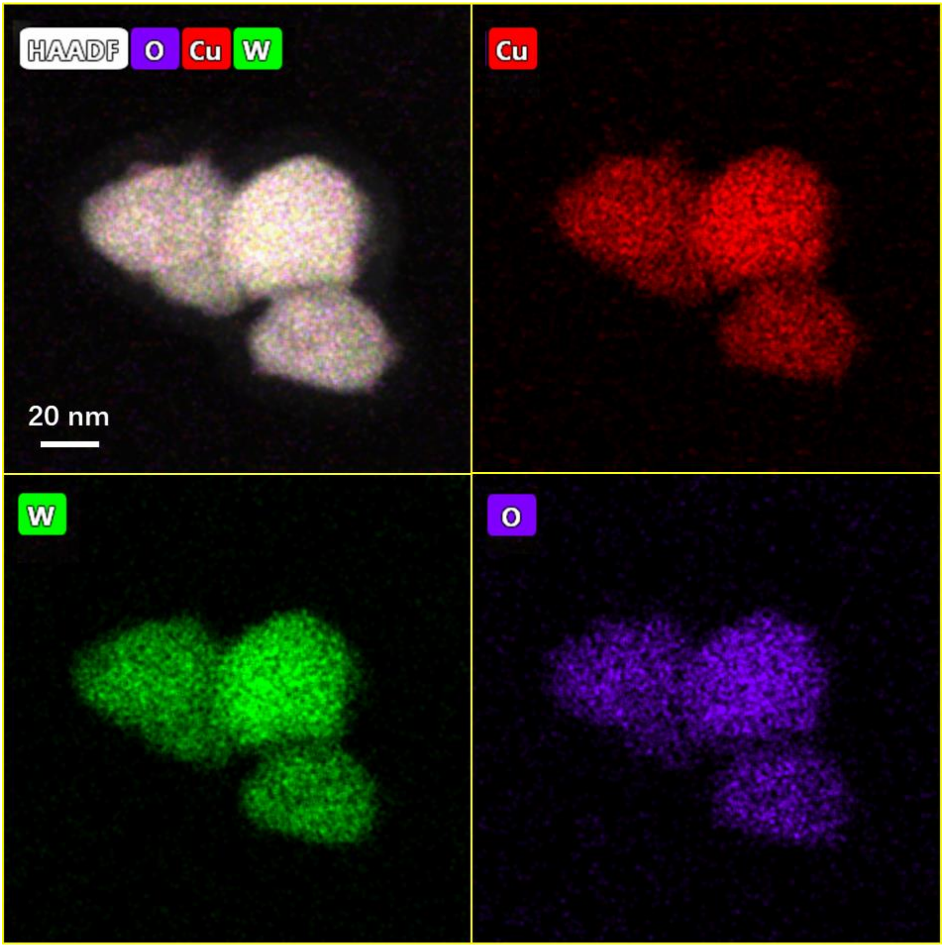

Supplementary Figure 3 HAADF and elemental mapping images of $CuWO_4$ catalyst.

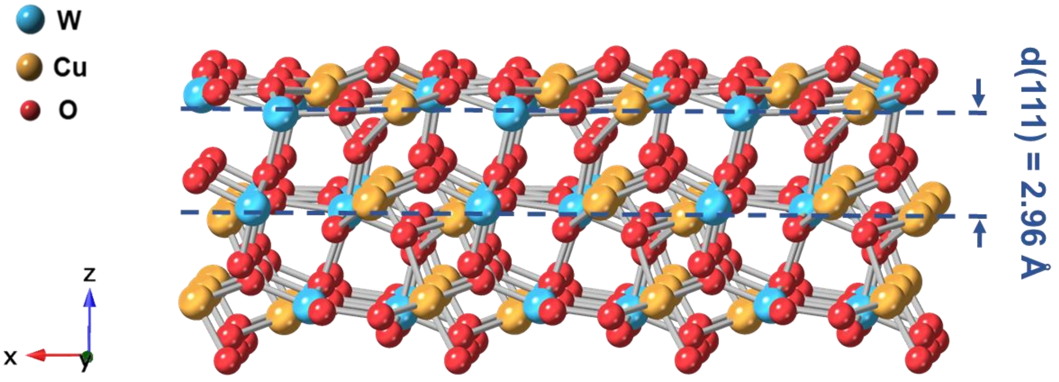

Supplementary Figure 4 The triclinic $CuWO_4$ crystal structure.

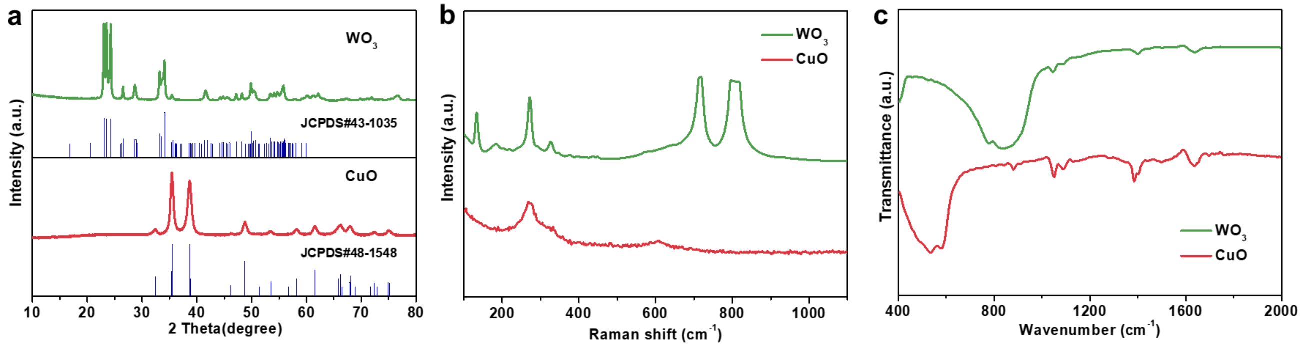

Supplementary Figure 5 (a) XRD patterns, (b) Raman spectra and (c) FT-IR spectra of WO_3 and CuO catalysts.

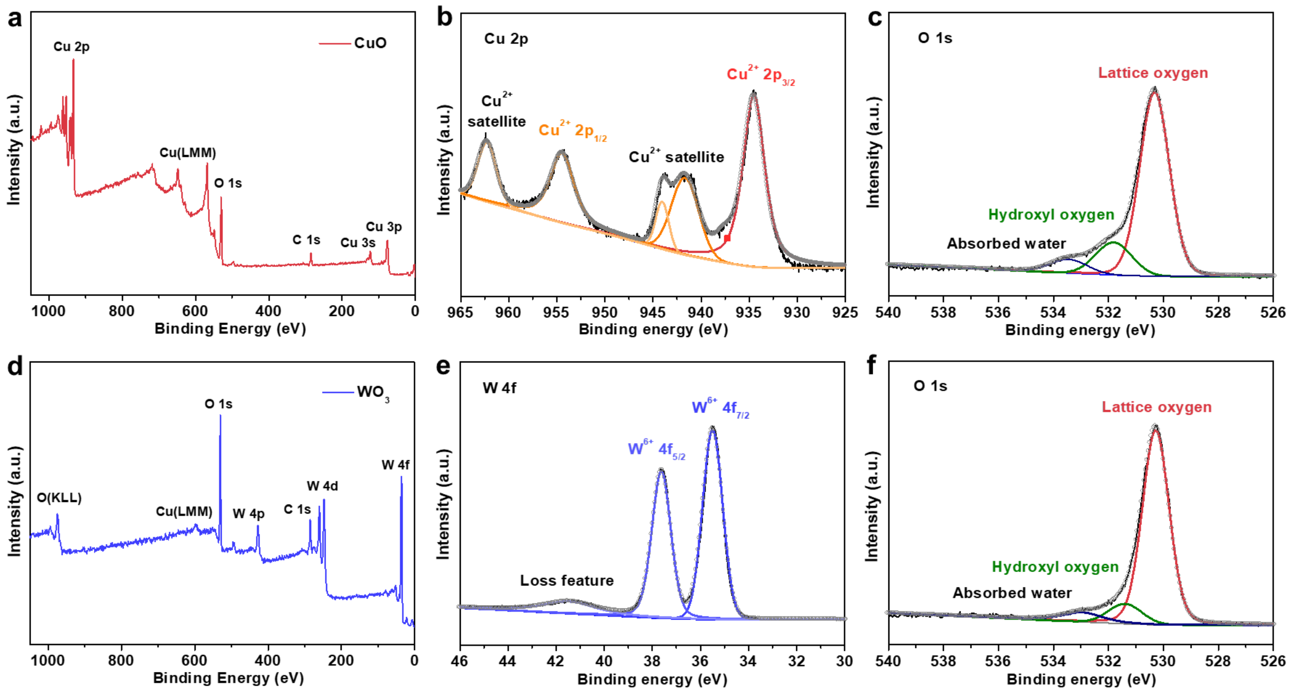

Supplementary Figure 6 (a) XPS survey spectra, (b) Cu 2p and (c) O 1s of CuO catalyst, and (d) XPS survey spectra, (e) W 4f and (f) O 1s of WO_3 catalyst.

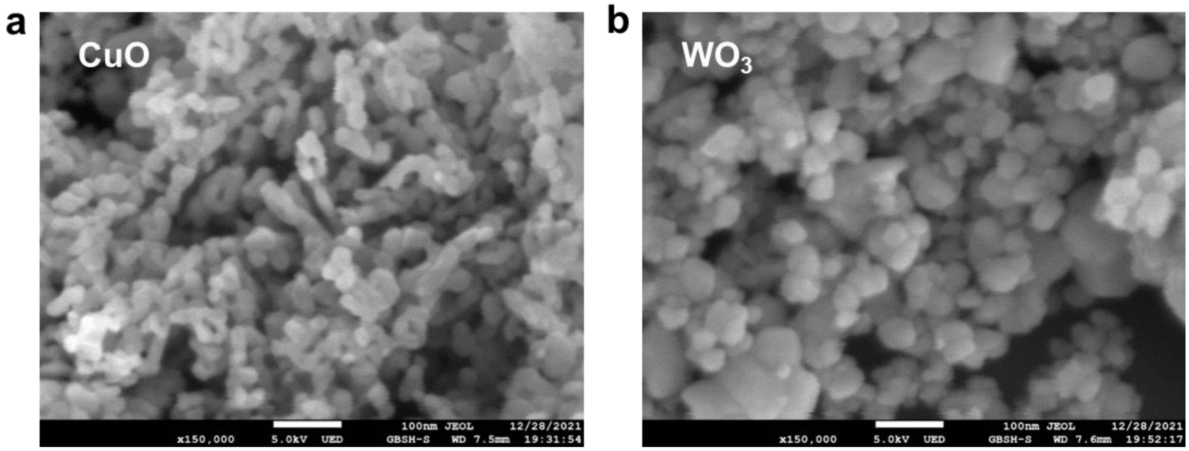

Supplementary Figure 7 SEM images of (a) CuO and (b) WO_3.

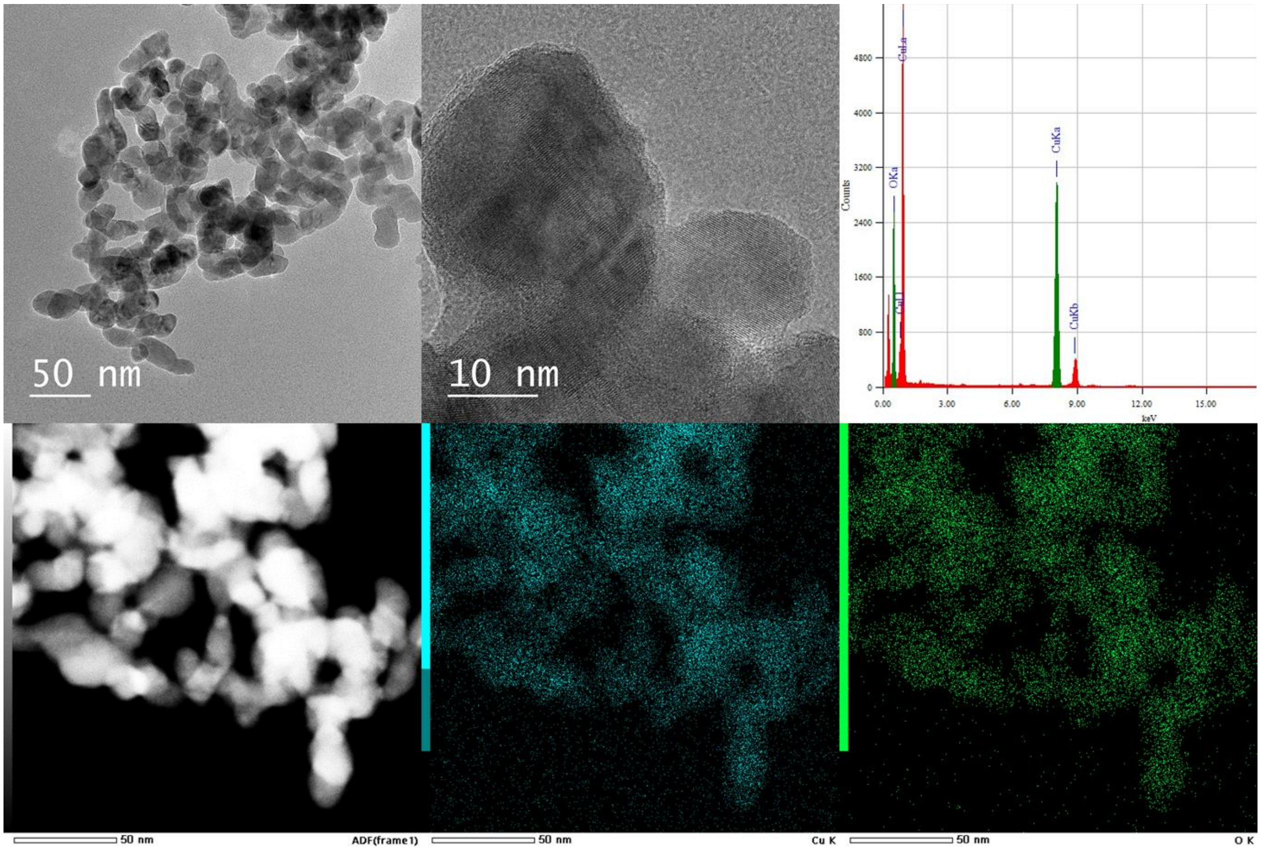

Supplementary Figure 8 TEM images, EDX energy spectrum, HAADF-STEM and elemental mappings images of CuO.

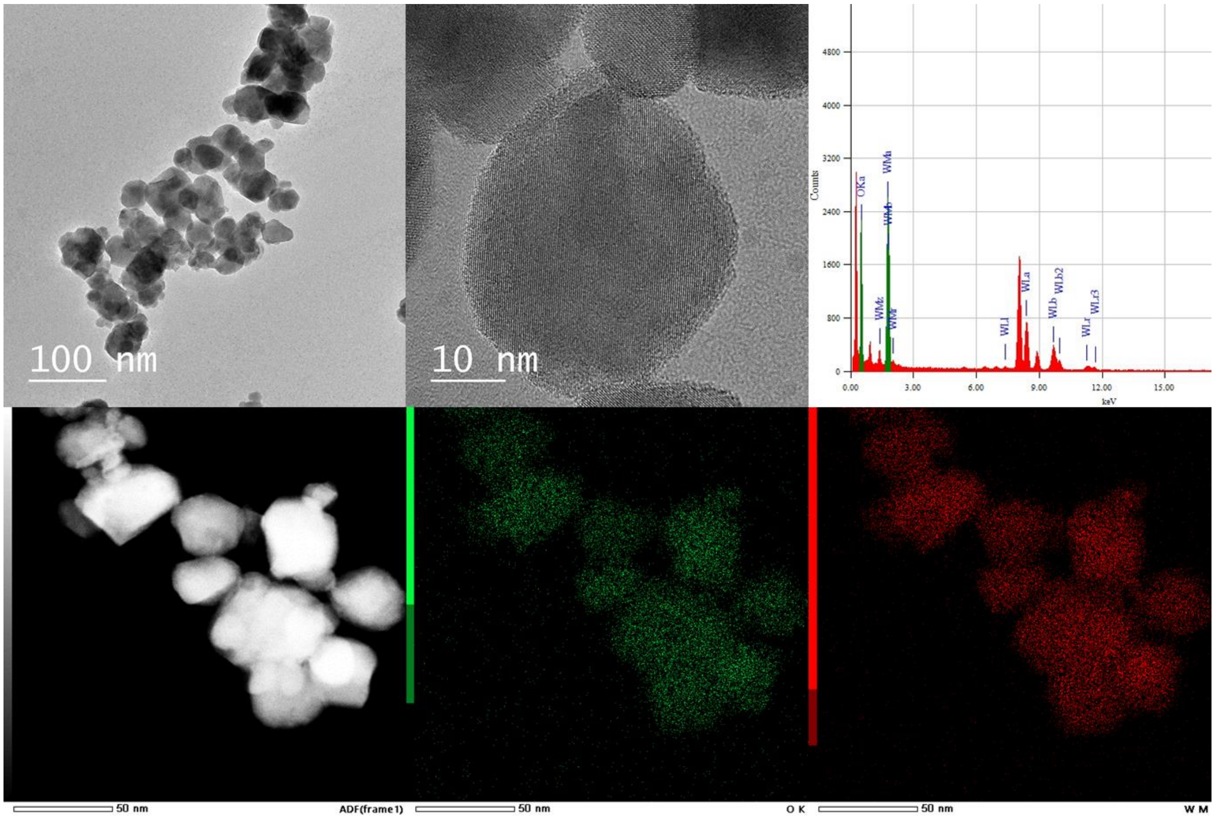

Supplementary Figure 9 TEM images, EDX energy spectrum, HAADF-STEM and elemental mappings images of WO_3.

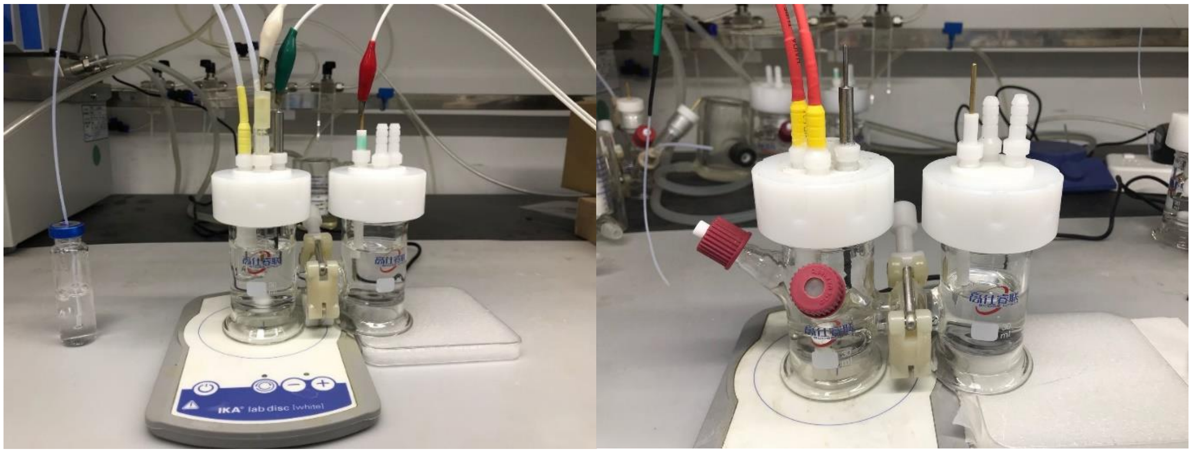

Supplementary Figure 10 The digital images of the H-cell and H-cell with sampling ports for electrochemical measurements.

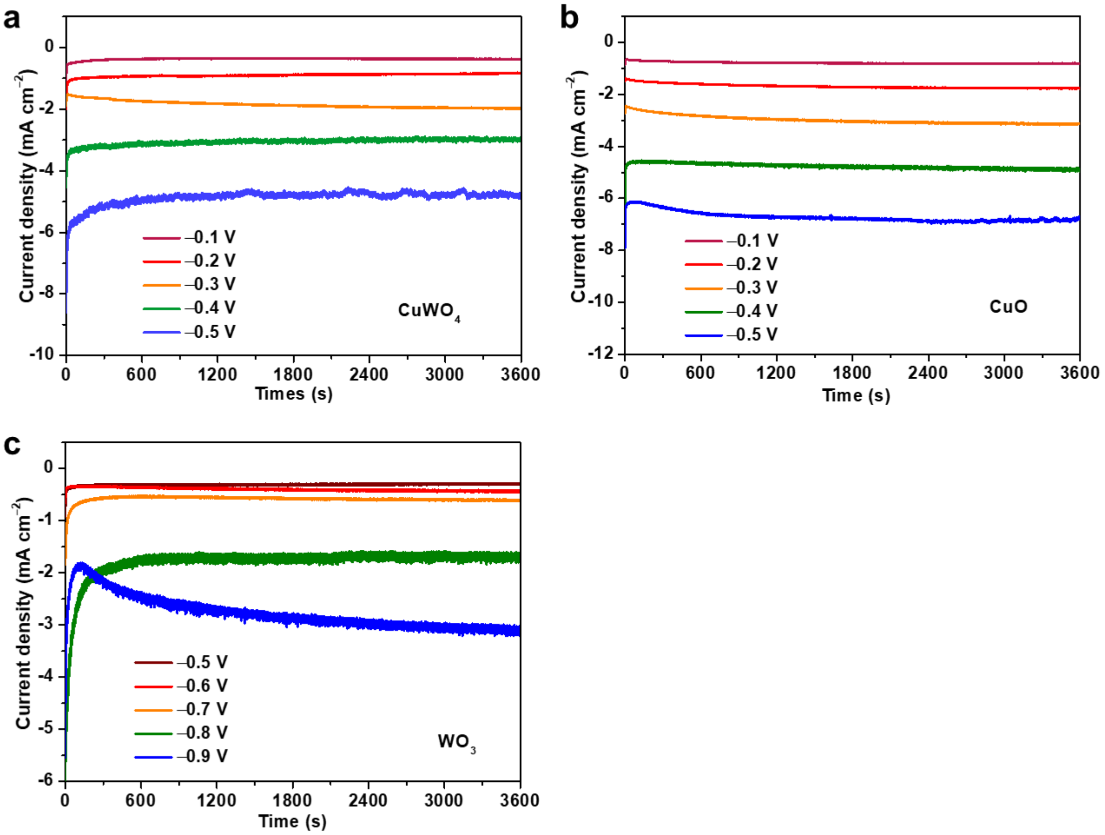

Supplementary Figure 11 Chrono-amperometry results of (a) $CuWO_4$, (b) CuO and (c) WO_3 in 0.1 M KNO_3 at the corresponding potentials.

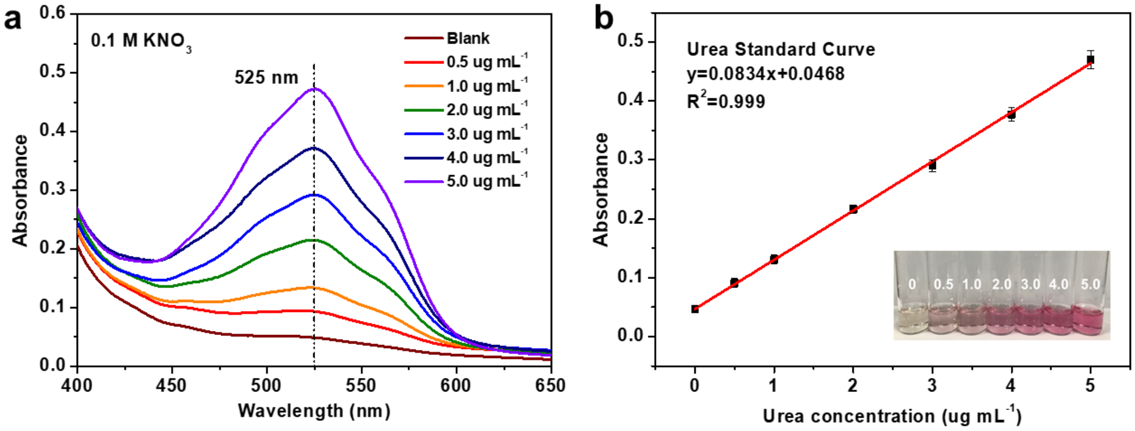

Supplementary Figure 12 Absolute calibration of the diacetylmonoxime method for quantification of urea. (a) UV-Vis spectra of urea with various concentrations. (b) The calibration curve for urea.

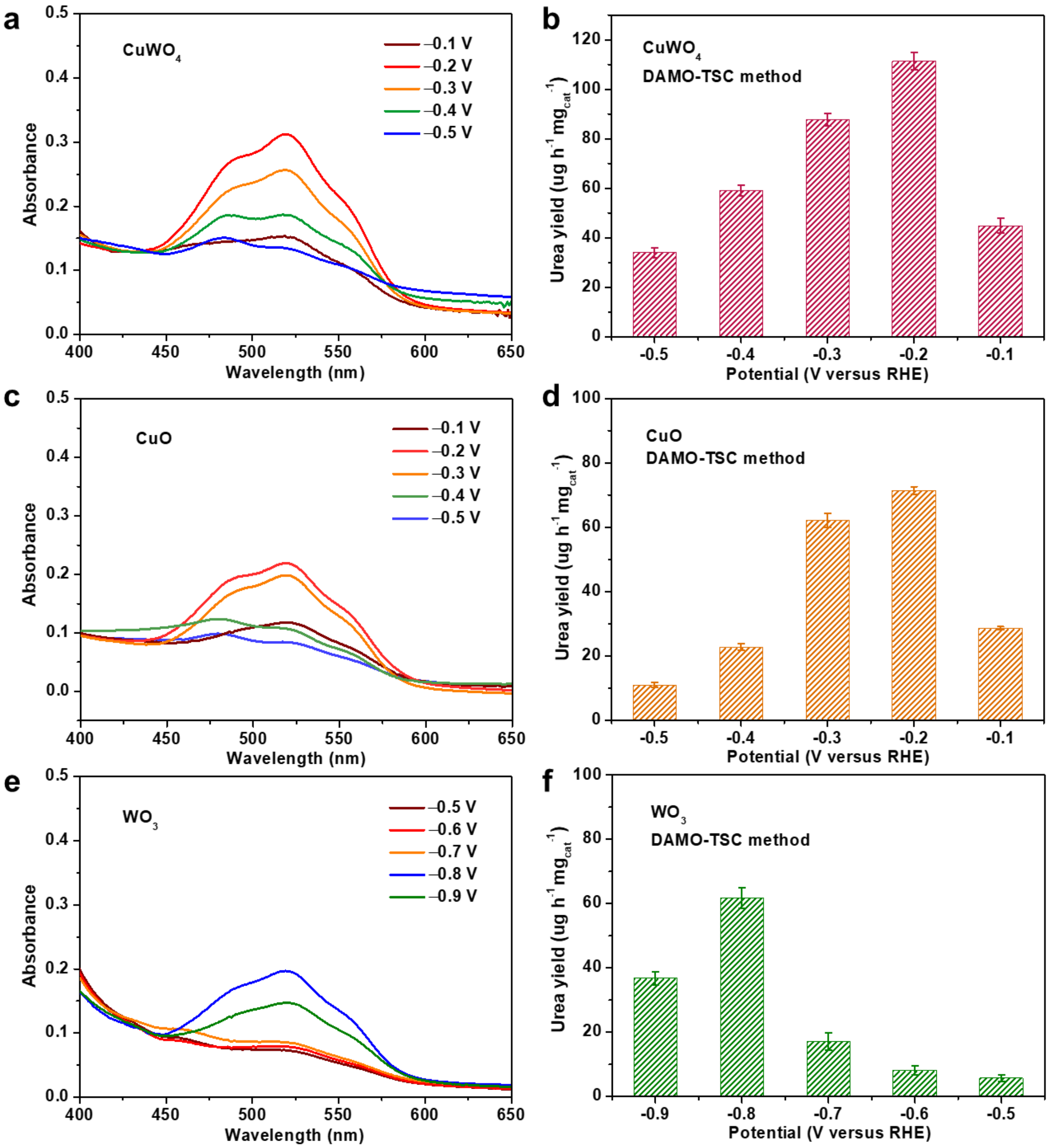

Supplementary Figure 13 Urea quantification of different catalysts electrolysis in 0.1 M KNO_3 electrolyte with CO_2 feeding gas at the corresponding potential for 1 hour. (a) UV-Vis spectra of the electrolyte of $CuWO_4$ with diacetylmonoxime indicator. (b) Urea yield rate at different potentials for $CuWO_4$. (c) UV-Vis spectra of the electrolyte of CuO with diacetylmonoxime indicator. (d) Urea yield rate at different potentials for CuO. (e) UV-Vis spectra of the electrolyte of WO_3 with diacetylmonoxime indicator. (f) Urea yield rate at different potentials for WO_3.

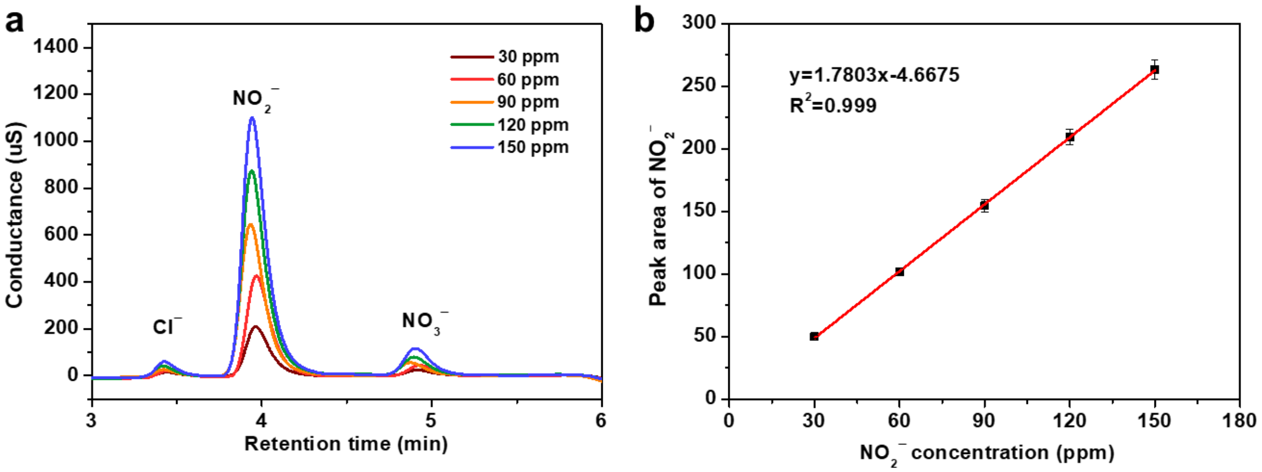

Supplementary Figure 14 Absolute calibration of ion chromatography for quantification of nitrite ions. (a) The measurement data of nitrite ions with various concentrations. (b) The corresponding standard curve of nitrite ions.

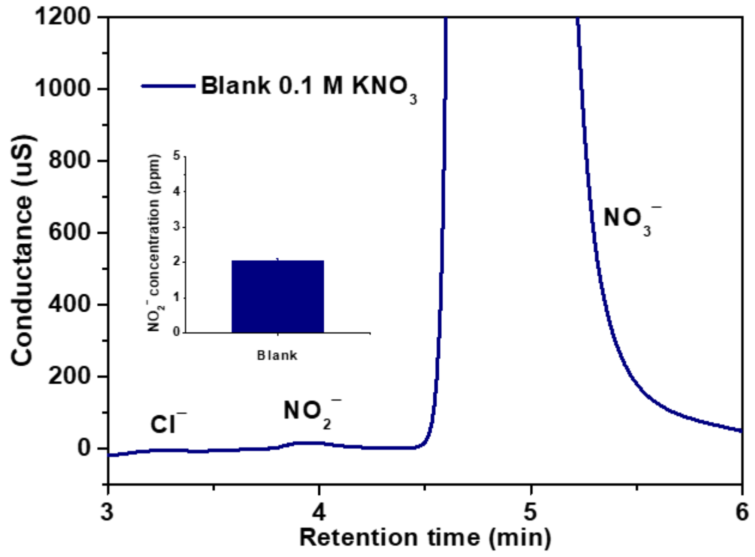

Supplementary Figure 15 Measurement data of nitrite ions in 0.1 M KNO_3 electrolyte by ion chromatography.

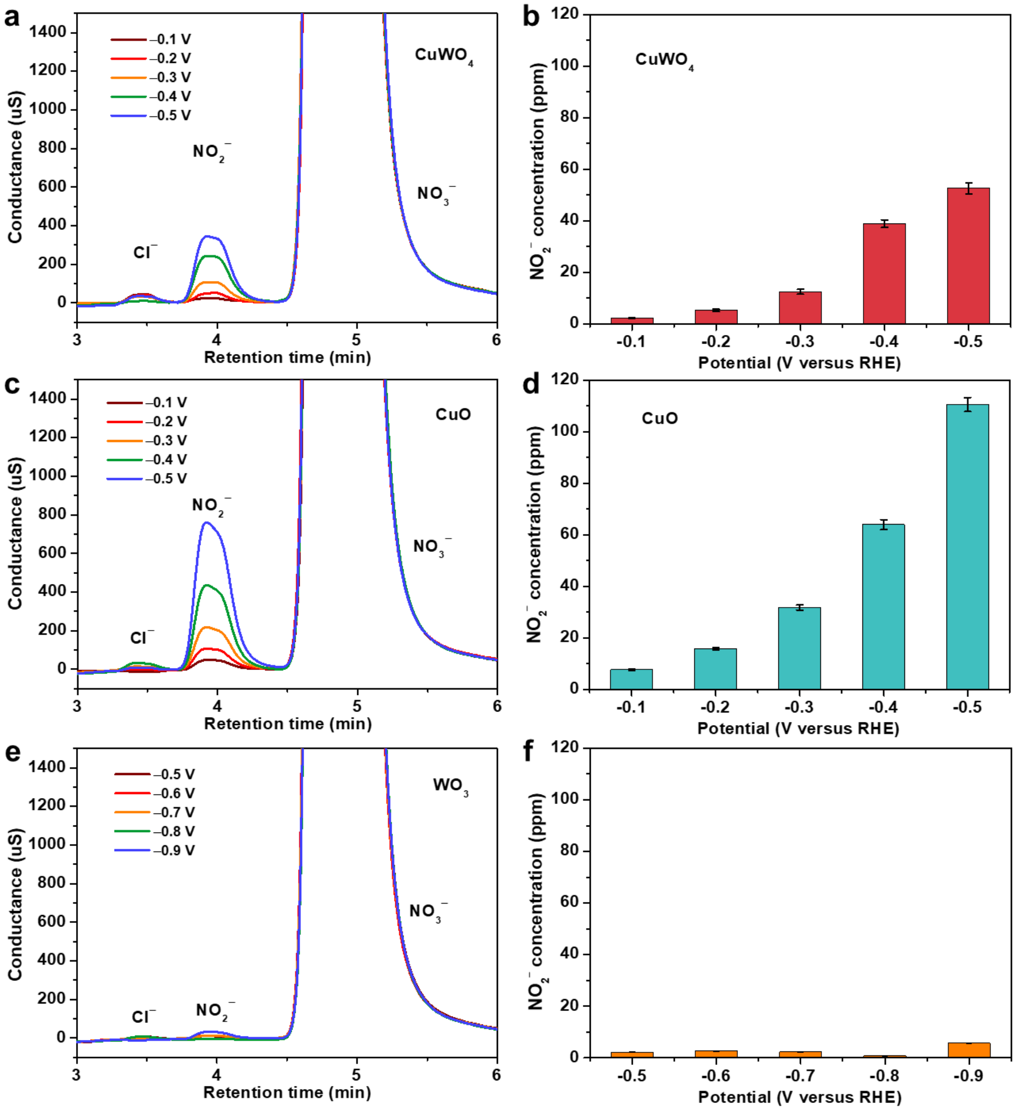

Supplementary Figure 16 Nitrite ions quantification of different catalysts electrolysis in 0.1 M KNO_3 electrolyte with CO_2 feeding gas at the corresponding potential for 1 hour. (a) The measurement datas of $CuWO_4$ electrolytes by ion chromatography. (b) Nitrite ion concentration at different potentials for $CuWO_4$. (c) The measurement datas of CuO electrolytes by ion chromatography. (d) Nitrite ion concentration at different potentials for CuO. (e) The measurement datas of WO_3 electrolytes by ion chromatography. (f) Nitrite ion concentration at different potentials for WO_3.

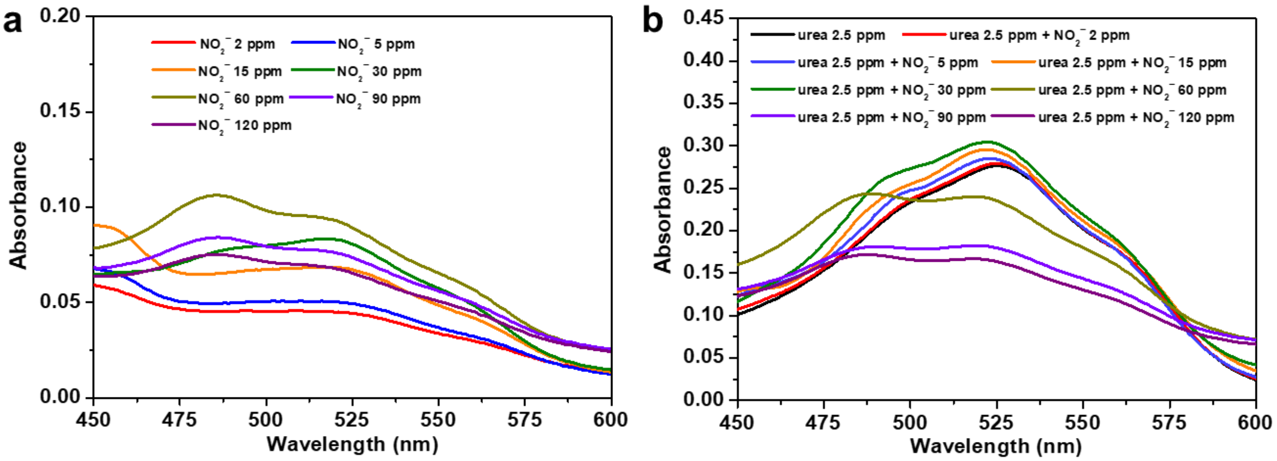

Supplementary Figure 17 Effect of co-existing nitrite on urea quantification by DAMO-TSC method. (a) UV-Vis spectra of nitrite standard solutions without urea by DAMO-TSC method. (b) UV-Vis spectra of nitrite standard solutions with 2.5 ppm urea by DAMO-TSC method.

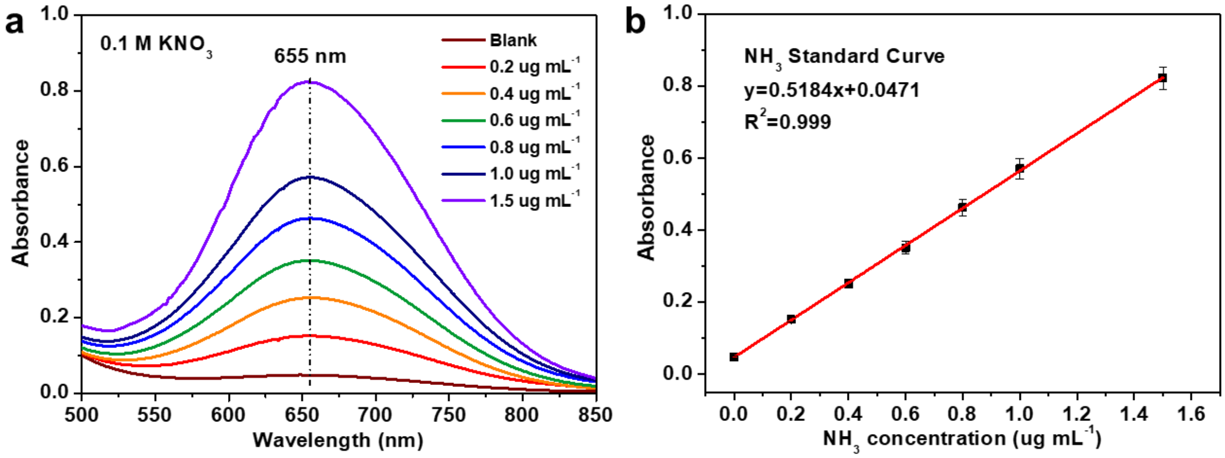

Supplementary Figure 18 Absolute calibration of the indophenol blue method for quantification of NH_3 estimated by NH_4^+ ion concentration. (a) UV-Vis spectra of NH_3 with various concentrations. (b) The calibration curve for NH_3.

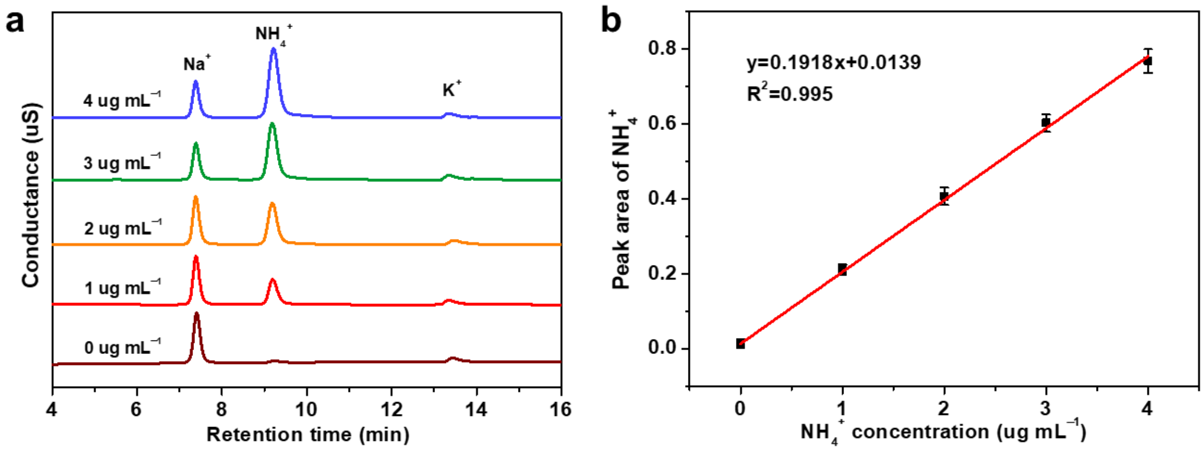

Supplementary Figure 19 Absolute calibration of the ion chromatography method for quantification of NH_4^+ ion concentration. (a) The chromatographic curves of NH_4^+ with various concentrations. (b) The calibration curve for NH_4^+.

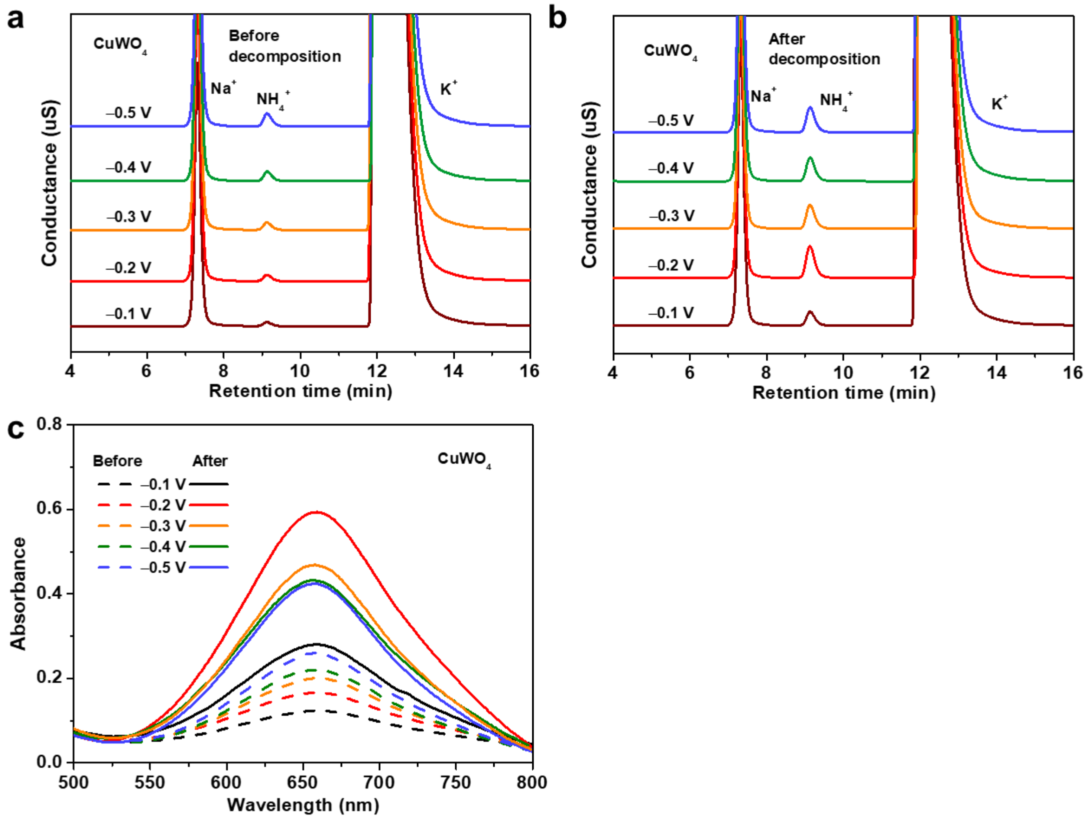

Supplementary Figure 20 Chromatographic curves (a, b) and UV-vis absorption spectra (c) of urea quantified by urease decomposition method for $CuWO_4$ based on ion chromatography and the indophenol blue method.

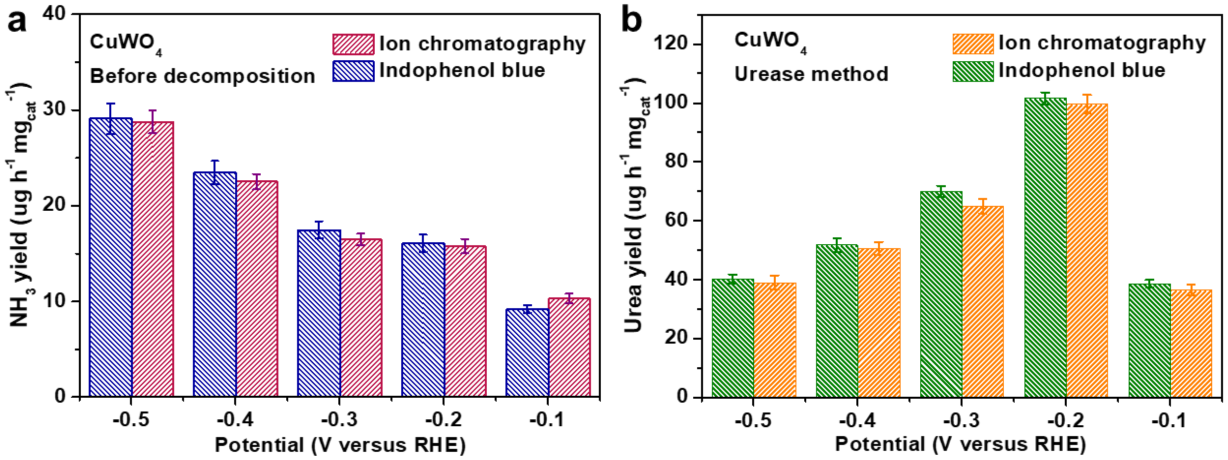

Supplementary Figure 21 (a) NH_3 yield rates of $CuWO_4$ via ion chromatography and the indophenol blue method. (b) Urea yield rates of $CuWO_4$ quantified by urease decomposition method based on ion chromatography and the indophenol blue method. (c) Faradaic efficiency of different products for $CuWO_4$ at different applied potentials.

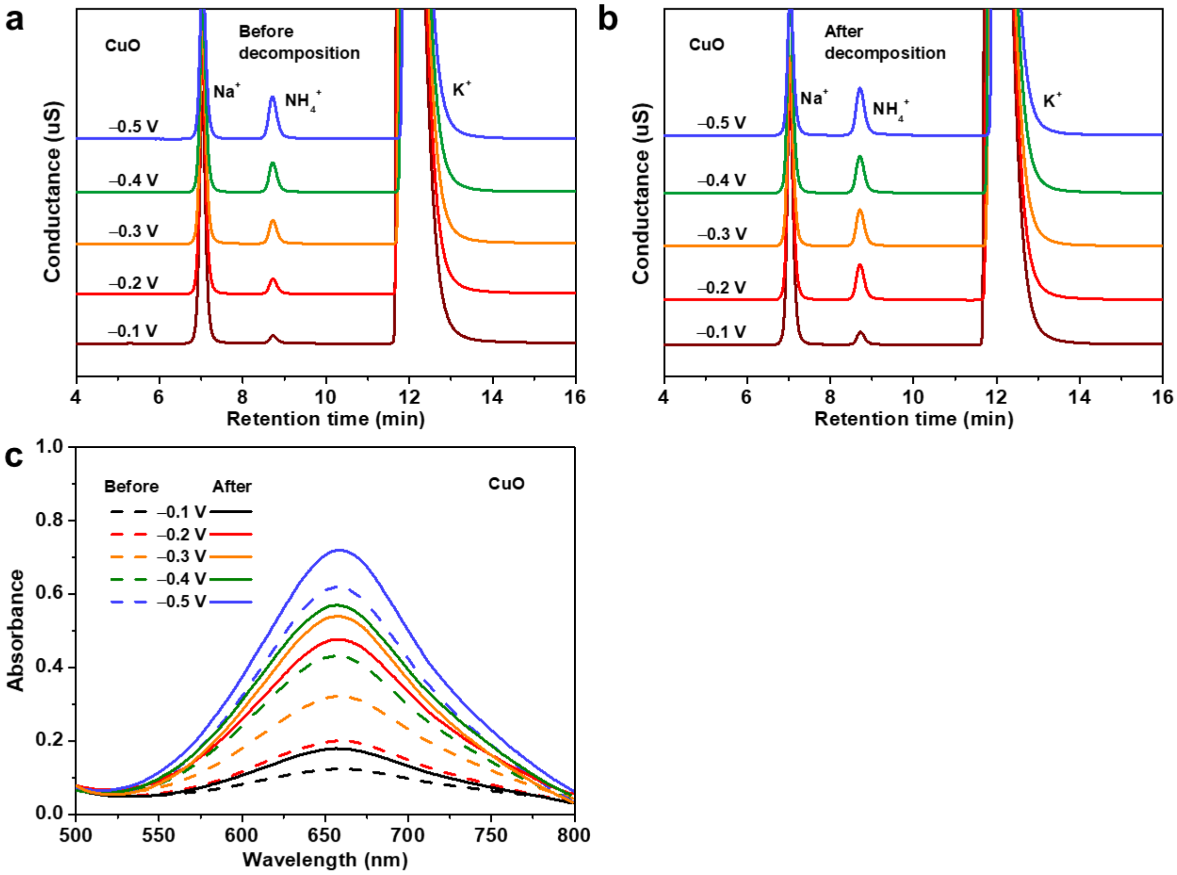

Supplementary Figure 22 Chromatographic curves (a, b) and UV-vis absorption spectra (c) of urea quantified by urease decomposition method for CuO based on ion chromatography and the indophenol blue method.

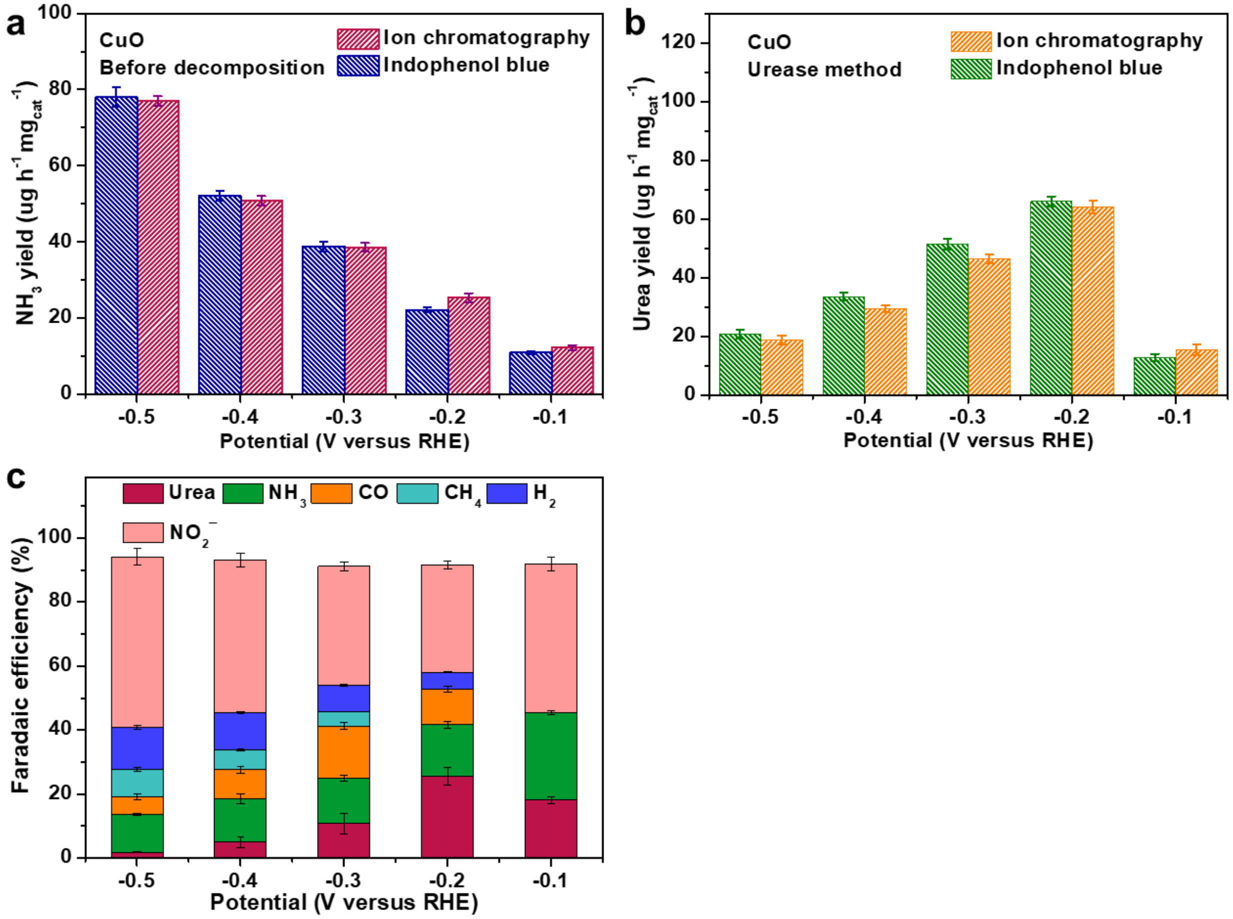

Supplementary Figure 23 (a) NH_3 yield rates of CuO via ion chromatography and the indophenol blue method. (b) Urea yield rates of CuO quantified by urease decomposition method based on ion chromatography and the indophenol blue method. (c) Faradaic efficiency of different products for CuO at different applied potentials.

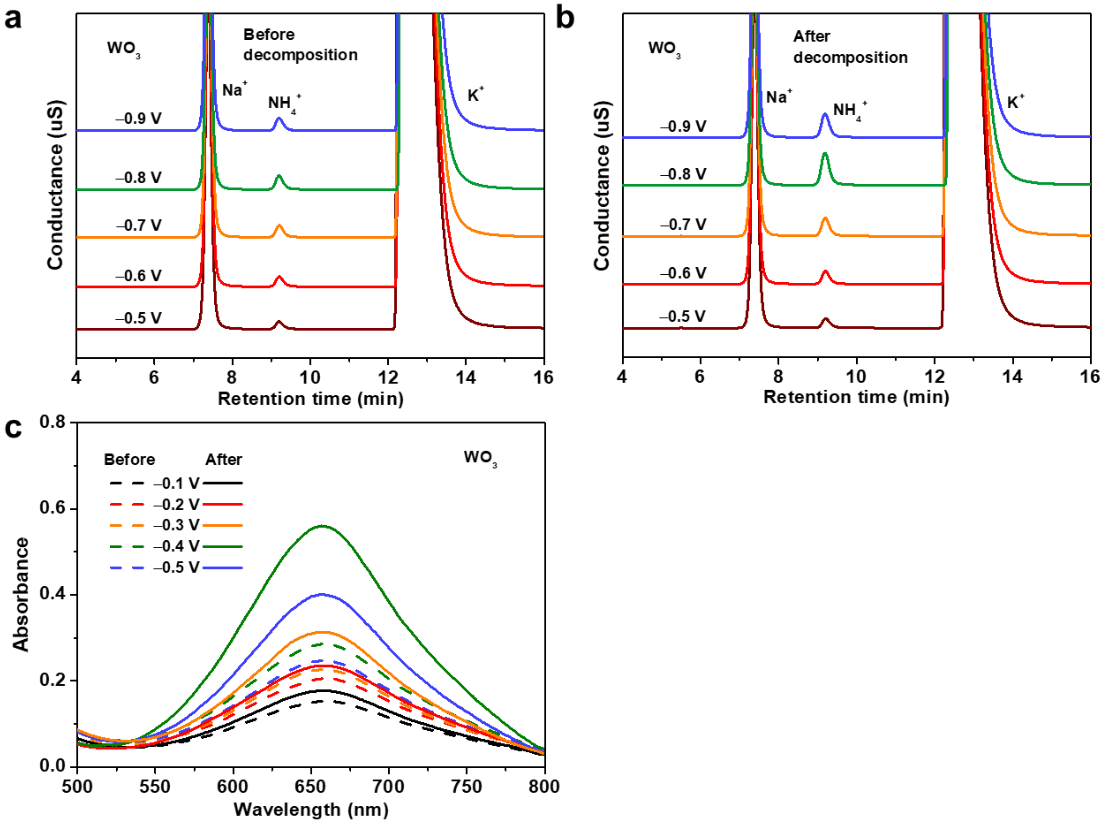

Supplementary Figure 24 Chromatographic curves (a, b) and UV-vis absorption spectra (c) of urea quantified by urease decomposition method for WO_3 based on ion chromatography and the indophenol blue method.

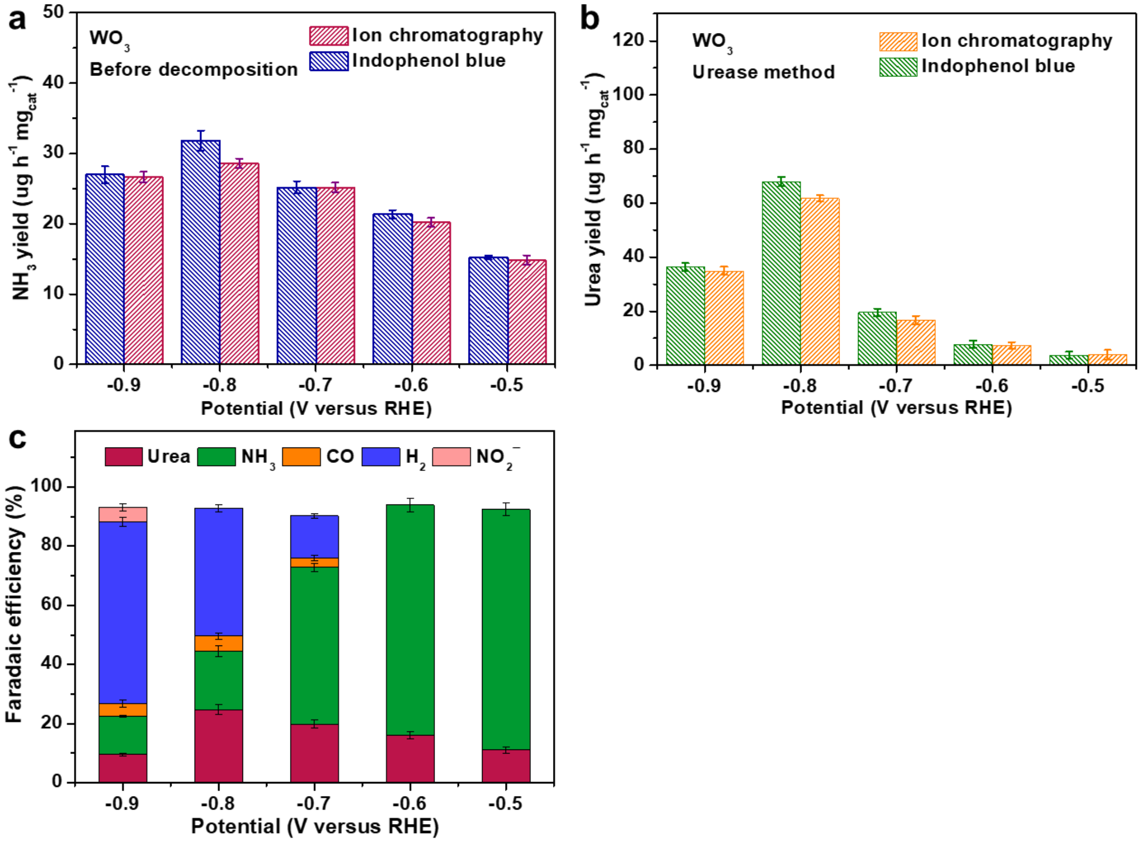

Supplementary Figure 25 (a) NH_3 yield rates of WO_3 via ion chromatography and the indophenol blue method. (b) Urea yield rates of WO_3 quantified by urease decomposition method based on ion chromatography and the indophenol blue method. (c) Faradaic efficiency of different products for WO_3 at different applied potentials.

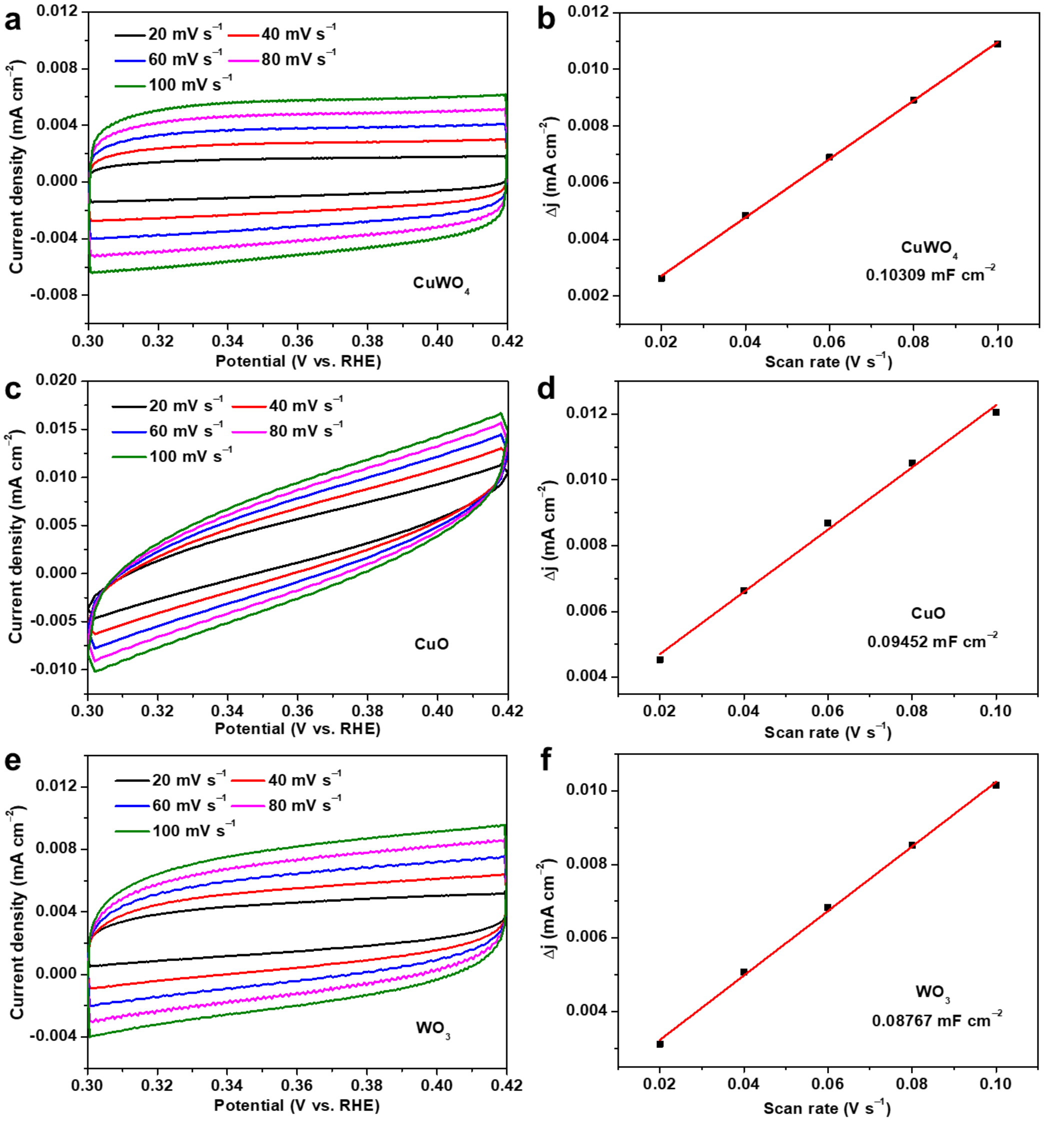

Supplementary Figure 26 Determination of the ECSA for the $CuWO_4$/CFP, CuO/CFP and WO_3/CFP electrodes. CV curves of (a) $CuWO_4$/CFP, (c) CuO/CFP and (e) WO_3/CFP in 0.1 M KNO_3 with different scan rates at selected potential range. The corresponding capacitance Δj($|j_{charge}-j_{discharge}|$) of (b) $CuWO_4$/CFP, (d) CuO/CFP and (f) WO_3/CFP electrodes versus the scan rates. The scanning potential range is from 0.3 V to 0.42 V vs RHE. ECSA of electrode was obtained from CV curves, in details, by plotting the Δj ($|j_{charge}-j_{discharge}|$) at Faradaic silence potential range against the scan rates, the linear slope is obtained, which is a positive correlation with the double-layer capacitance (Cdl), and been used to represent the corresponding ECSA.

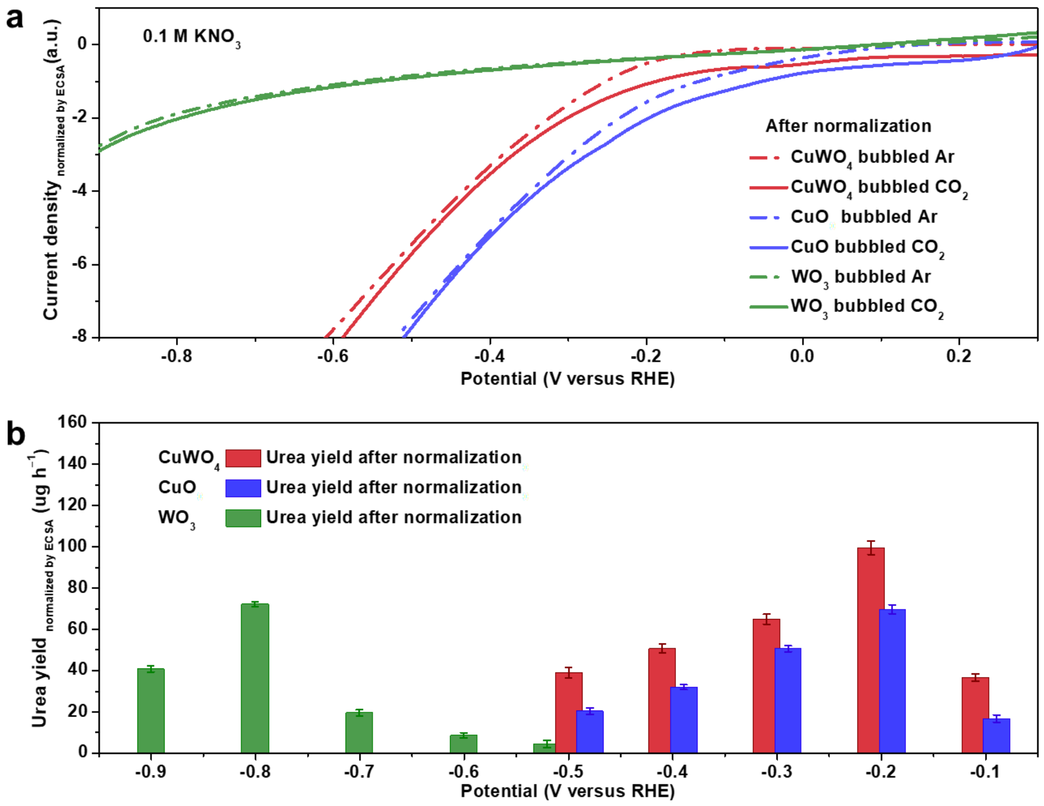

Supplementary Figure 27 (a) The LSV curves and (b) urea yield normalized by ECSA of $CuWO_4$/CFP, CuO/CFP and WO_3/CFP electrodes.

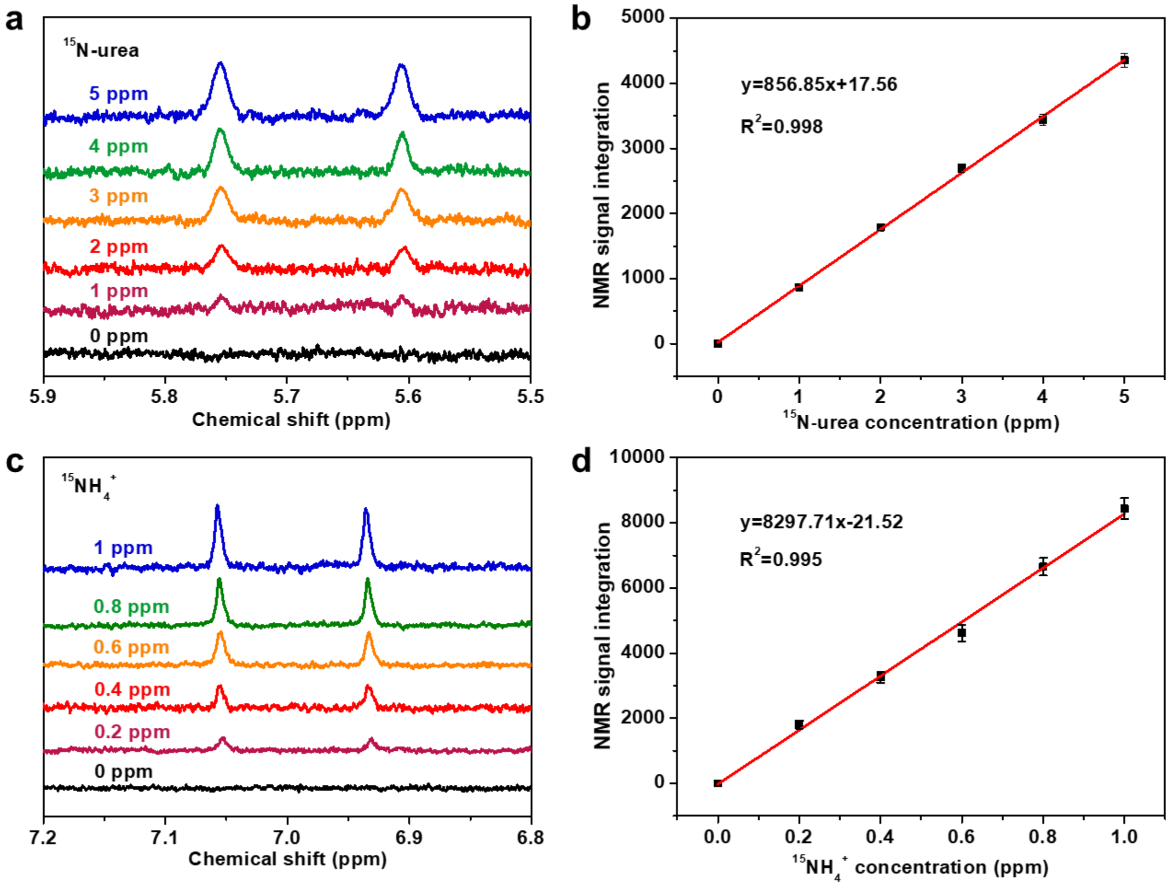

Supplementary Figure 28 NMR spectra and calibration curves for quantification of ^{15}N-urea and $^{15}NH_4^+$. (a) NMR spectra of ^{15}N-urea with various concentrations. (b) The calibration curves for ^{15}N-urea. (c) NMR spectra of $^{15}NH_4^+$ with various concentrations. (d) The calibration curves for $^{15}NH_4^+$.

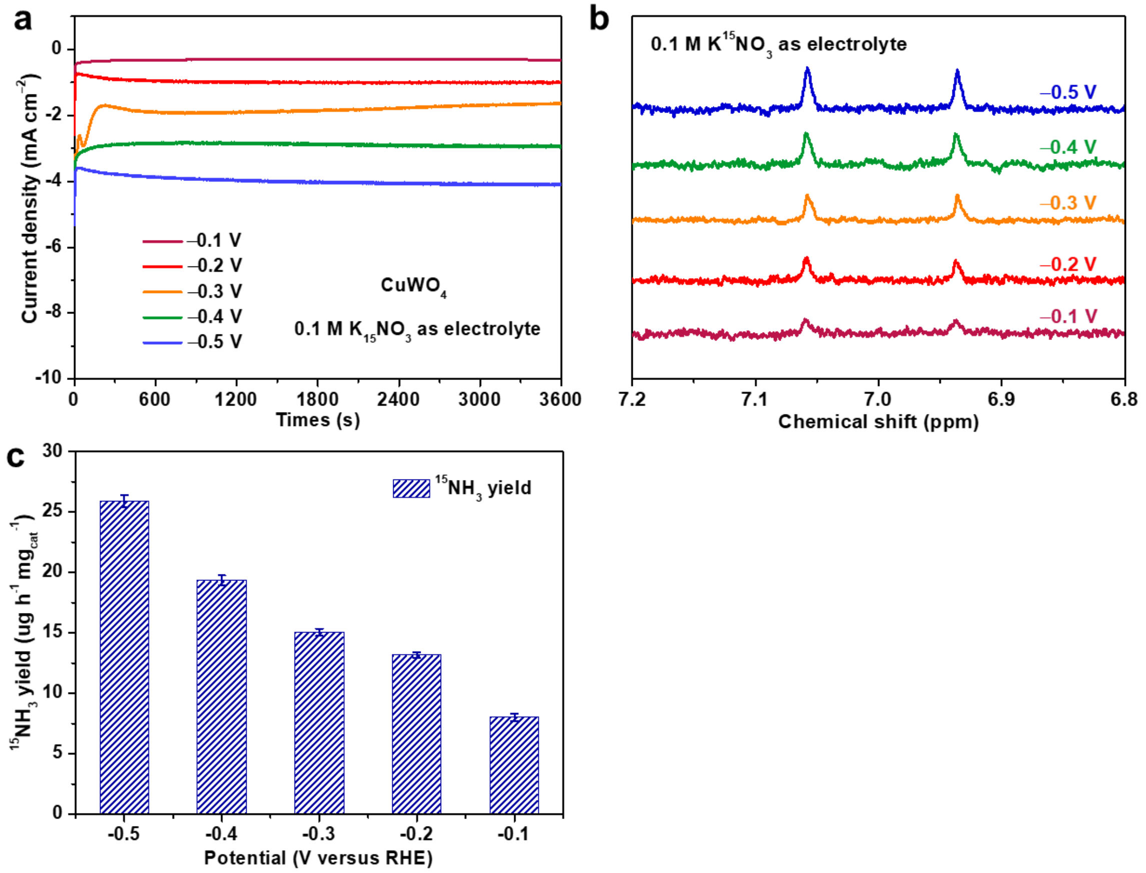

Supplementary Figure 29 (a) Chrono-amperometry results of $CuWO_4$ in 0.1 M $K^{15}NO_3$ with CO_2 feeding at the corresponding potentials. (b) ^1H NMR data of isotope calibration experiment in 0.1 M $K^{15}NO_3$ with CO_2 bubbling (20 mL min^{-1}) at different applied potentials. (c) $^{15}NH_3$ yield rates via integrated peak area from NMR data.

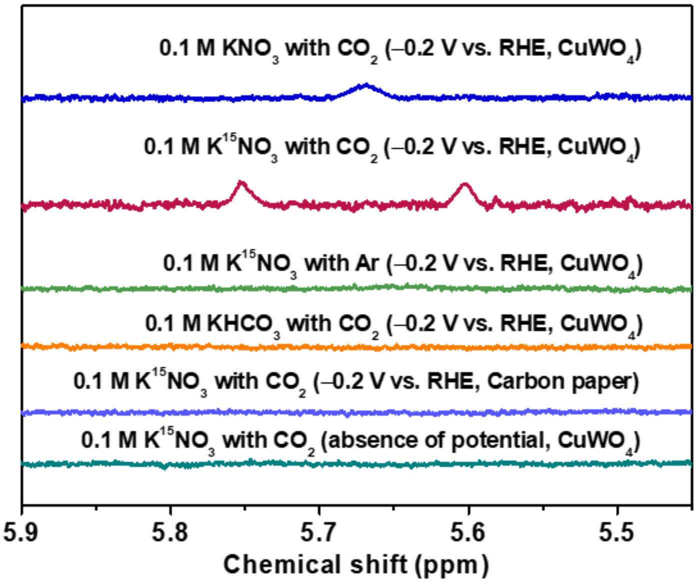

Supplementary Figure 30 NMR spectroscopy results of $CuWO_4$ in 0.1 M KNO_3 with CO_2 at −0.2 V versus RHE, $CuWO_4$ in 0.1 M $K^{15}NO_3$ with CO_2 at −0.2 V versus RHE and other control experiments.

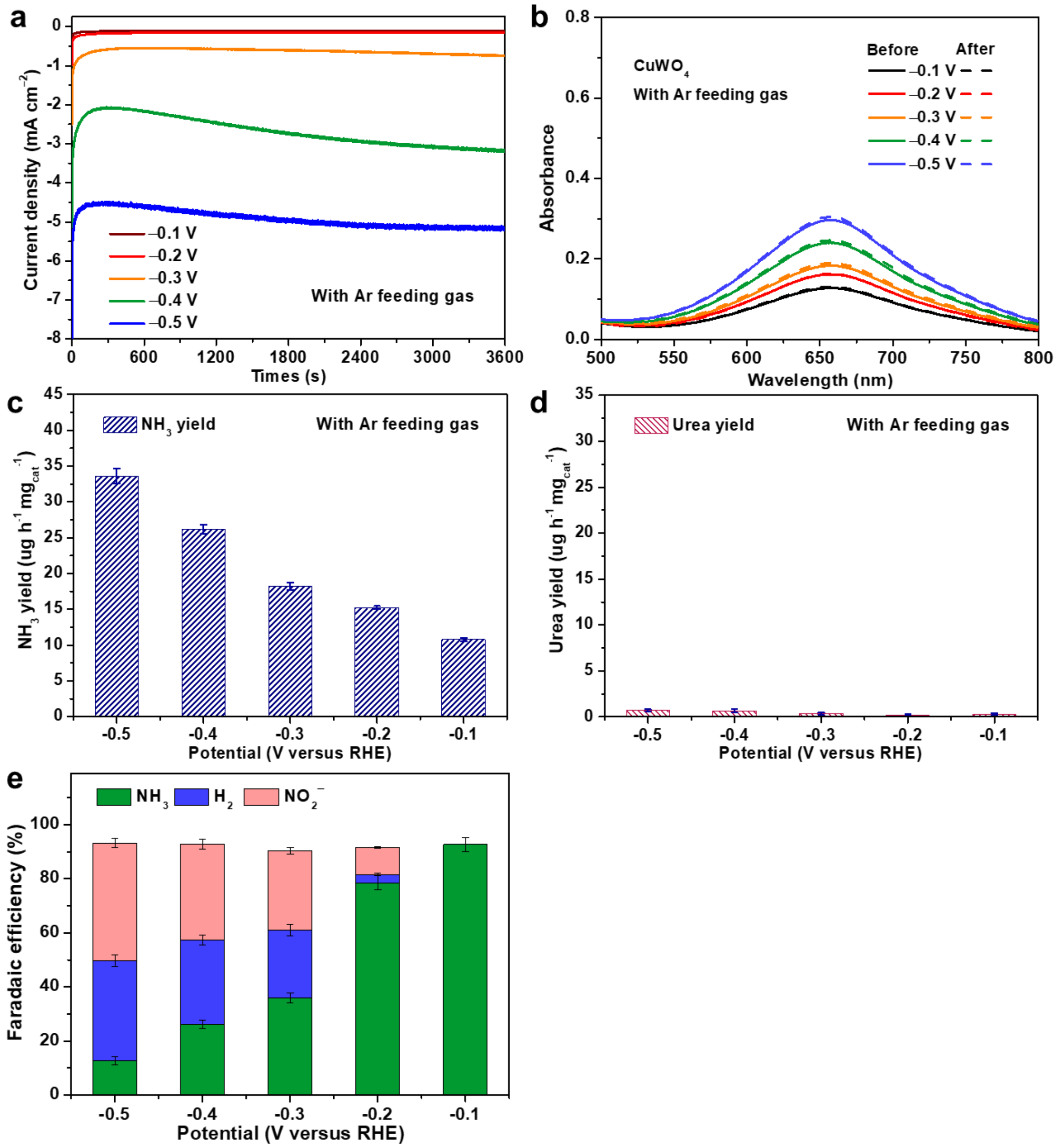

Supplementary Figure 31 $CuWO_4$ electrolysis in 0.1 M KNO_3 electrolyte with Ar bubbling at the corresponding potential for 1 hour. (a) Chrono-amperometry results of $CuWO_4$ at the corresponding potentials. (b) UV-Vis spectra of the electrolyte before and after urease decomposition with indophenol indicator. (c) NH_3 yield rate at different potentials for $CuWO_4$. (d) Urea yield rate on $CuWO_4$ at different applied potentials. (e) Faradaic efficiency of different products for $CuWO_4$ at different applied potentials.

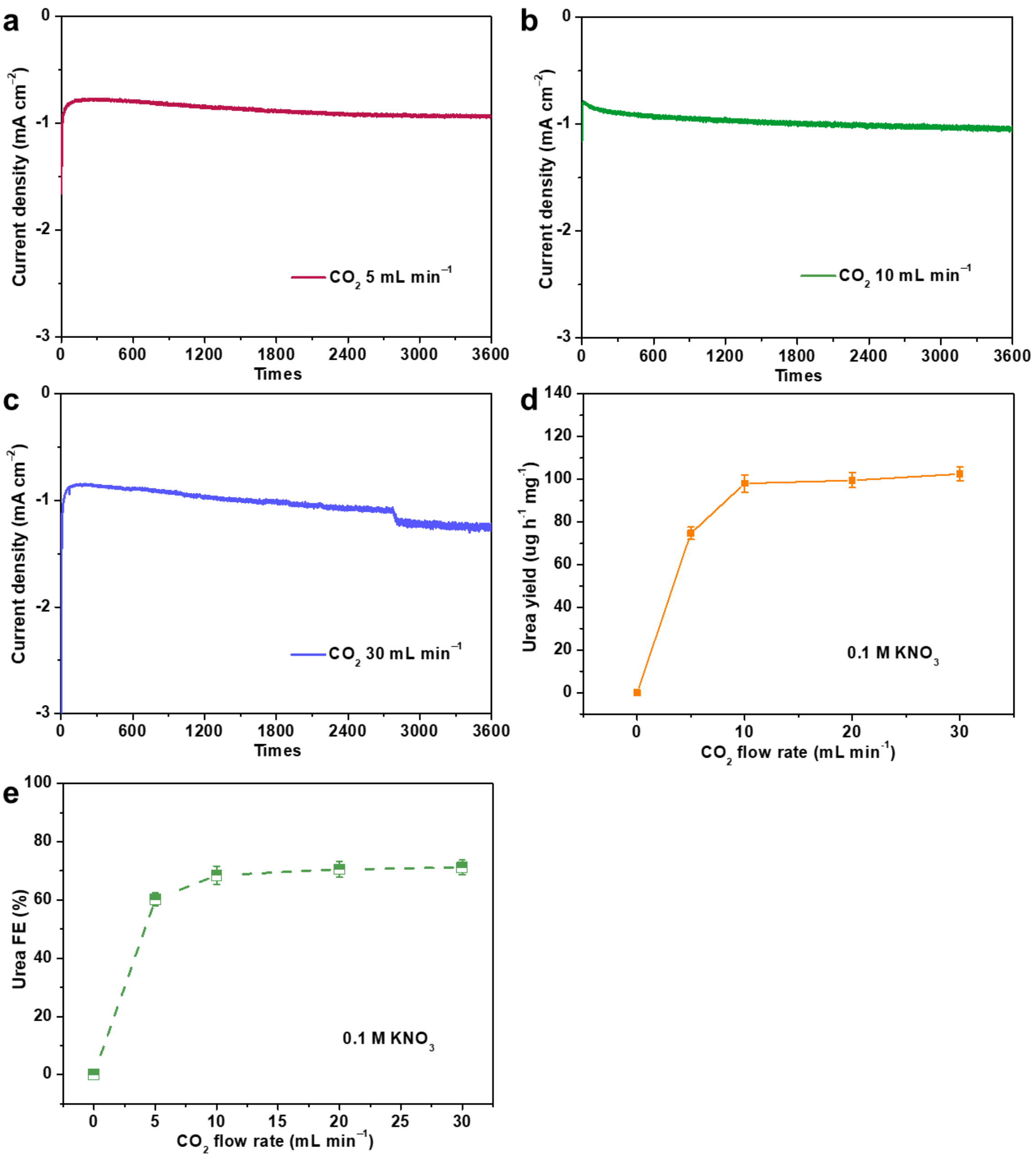

Supplementary Figure 32 Chrono-amperometry results of $CuWO_4$ at −0.2 V versus RHE in 0.1 M KNO_3 with (a) 5 mL min^{-1}, (b) 10 mL min^{-1} and (c) 30 mL min^{-1} CO_2 feeding flow rates. (d) Urea yield rates for $CuWO_4$ at −0.2 V versus RHE in 0.1 M KNO_3 with different CO_2 feeding flow rates. (e) Urea FEs for $CuWO_4$ with different CO_2 feeding flow rates.

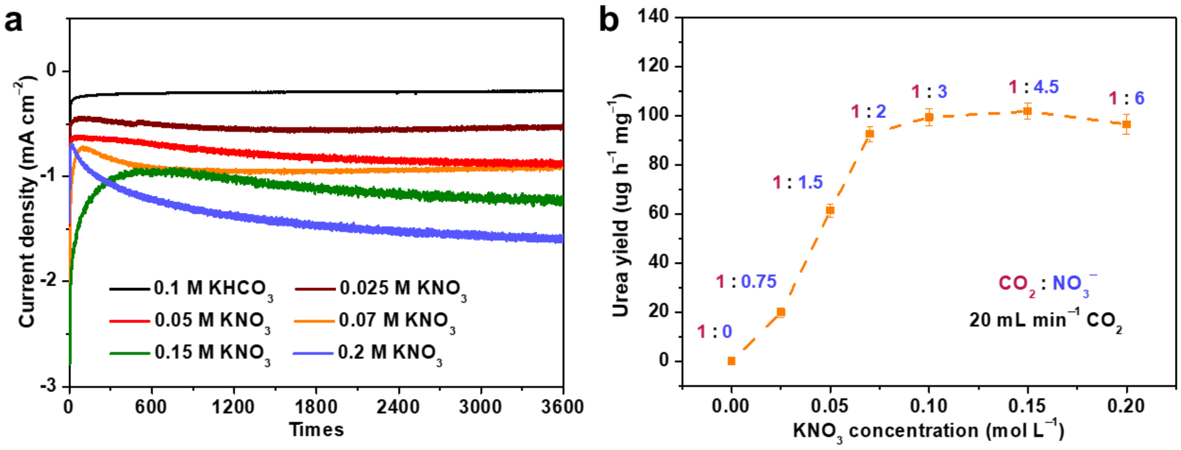

Supplementary Figure 33 (a) Chrono-amperometry results and (b) urea yield rates of $CuWO_4$ at −0.2 V versus RHE in different concentrations of KNO_3 electrolyte with CO_2 bubbling (20 mL min^{-1}).

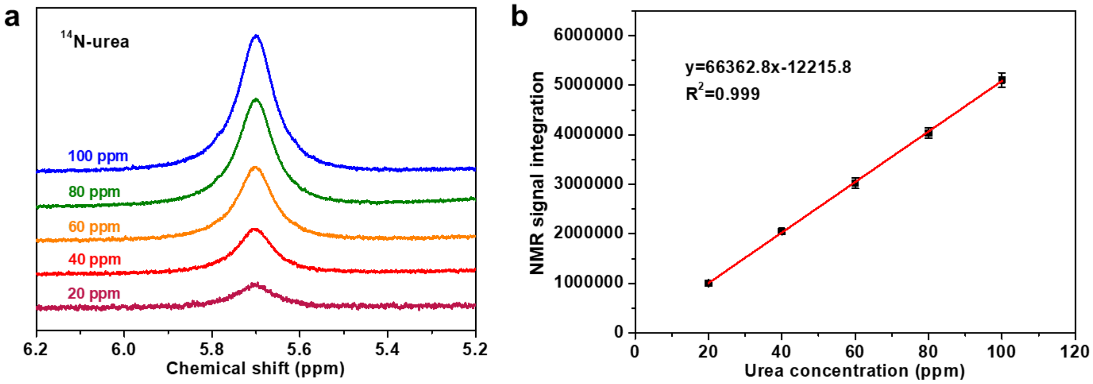

Supplementary Figure 34 (a) ^{1}H NMR spectra of ^{14}N-urea with various concentrations. (b) The calibration curves for ^{14}N-urea.

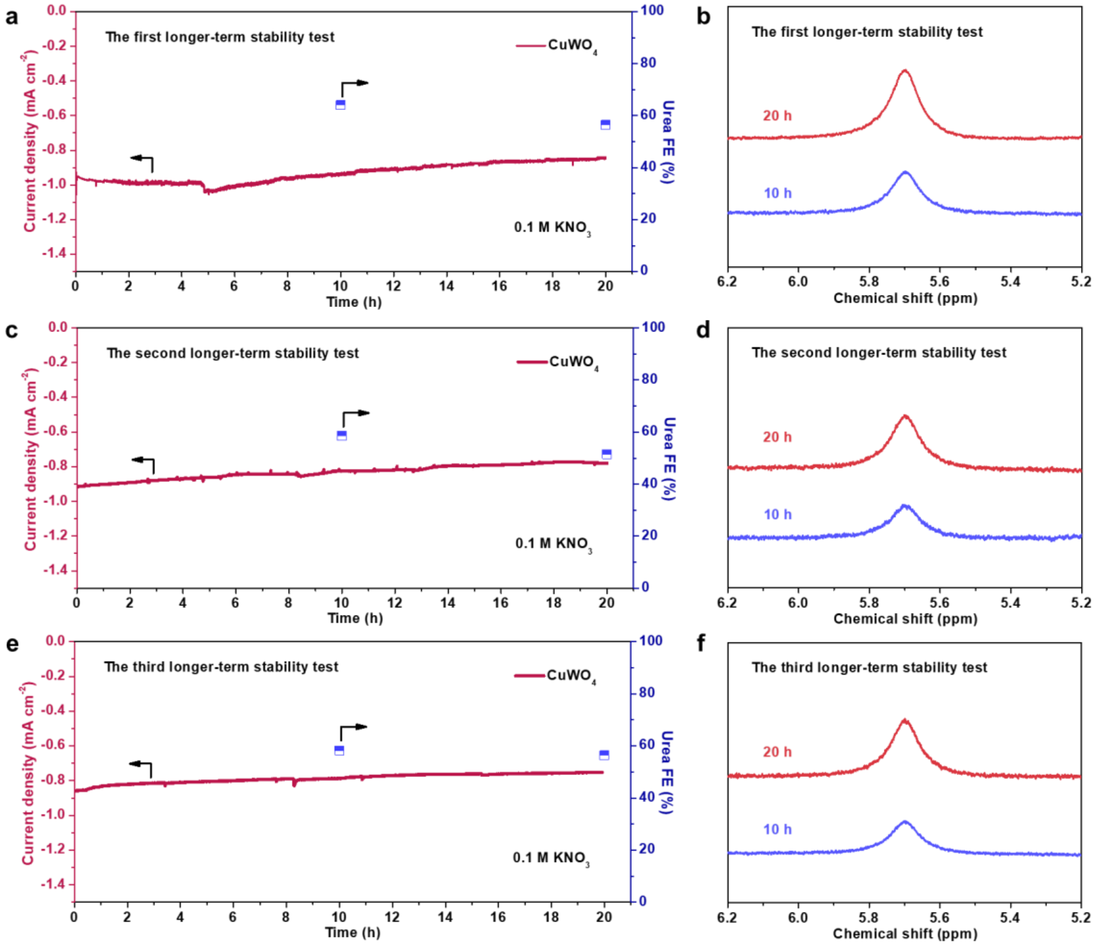

Supplementary Figure 35*.* (a, c, e) three long-term repeated tests of urea synthesis during 20 h of electrolysis at −0.2 V vs. RHE in 0.1 M KNO_3 with CO_2 bubbling (20 mL min^{-1}) and (b, d, f) ^1H NMR data of corresponding electrolyte.

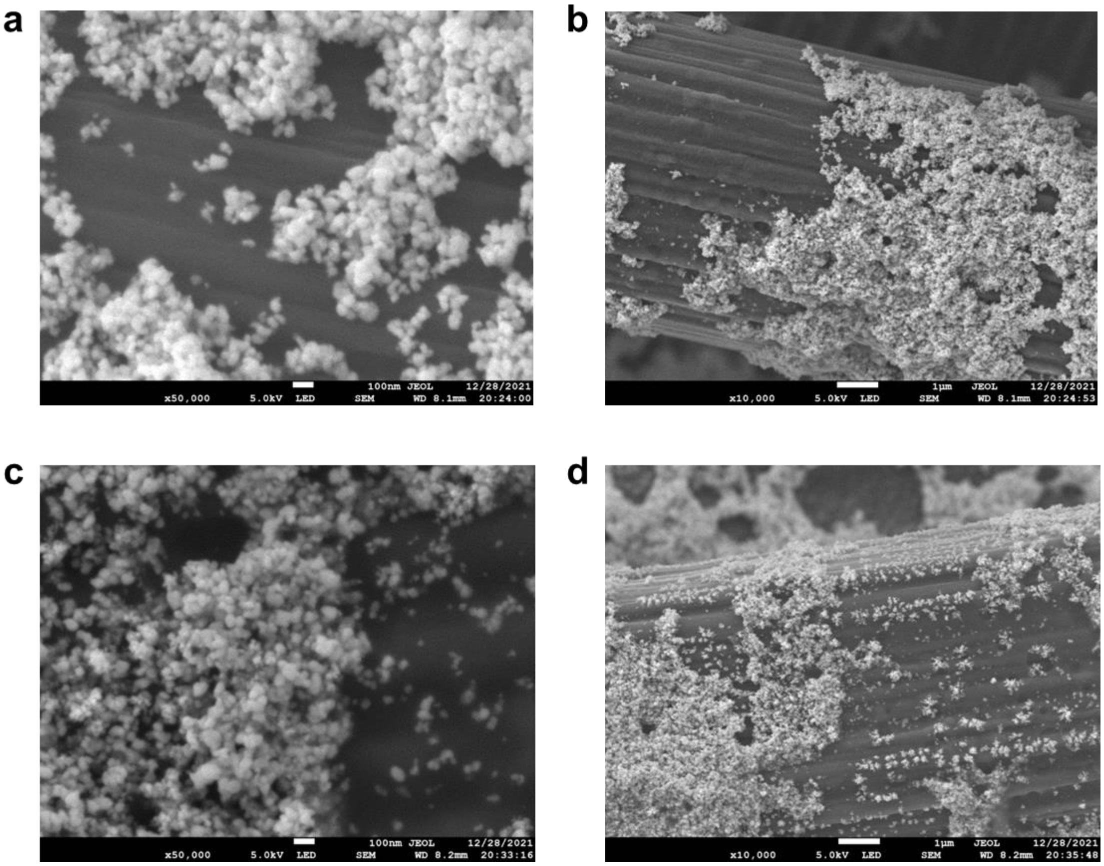

Supplementary Figure 36 (a,b) SEM images of $CuWO_4$/CP electrode before catalysis reaction. (c,d) SEM images of $CuWO_4$/CP electrode after catalysis reaction.

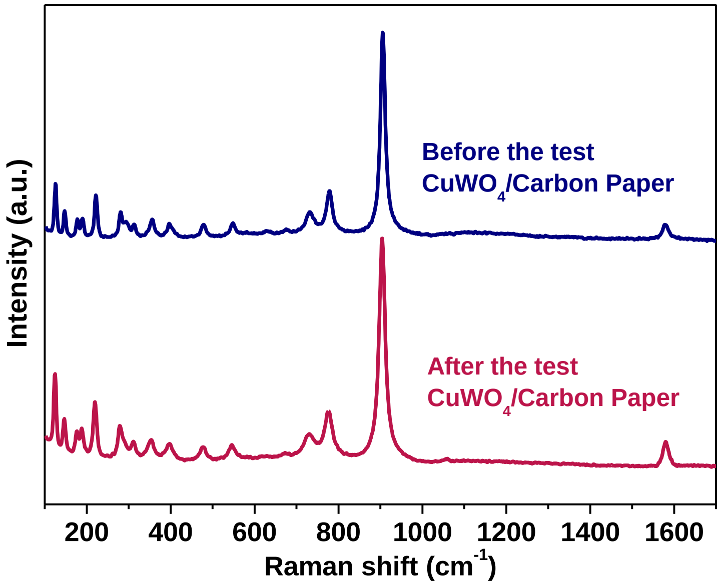

Supplementary Figure 37 Raman spectra of $CuWO_4$/CP electrodes before and after catalysis reaction.

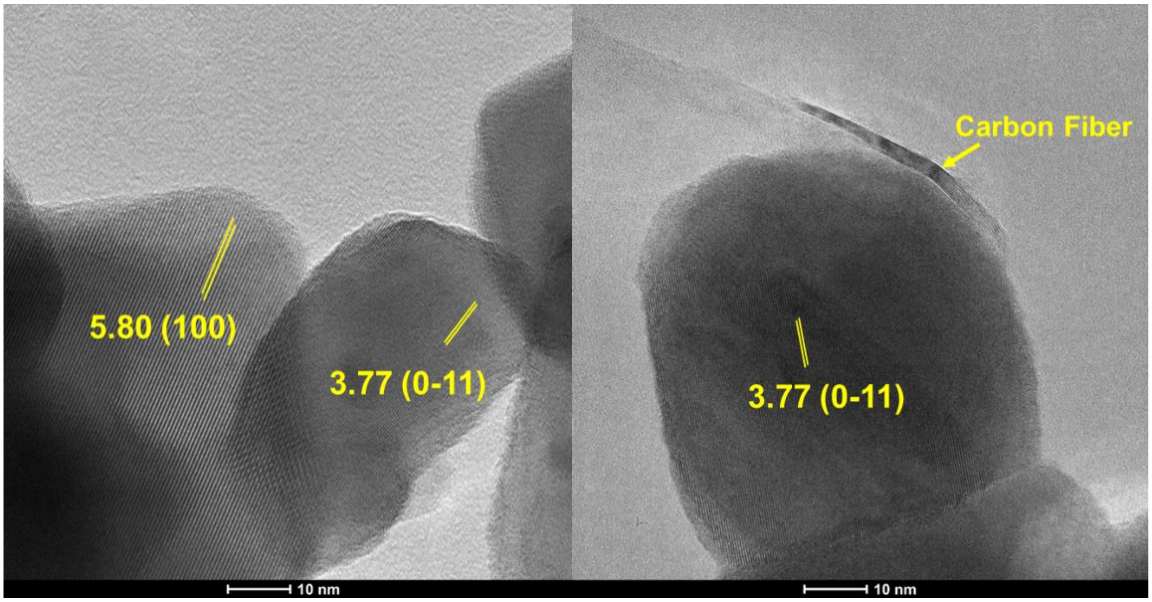

Supplementary Figure 38 TEM images of $CuWO_4$ after catalysis reaction.

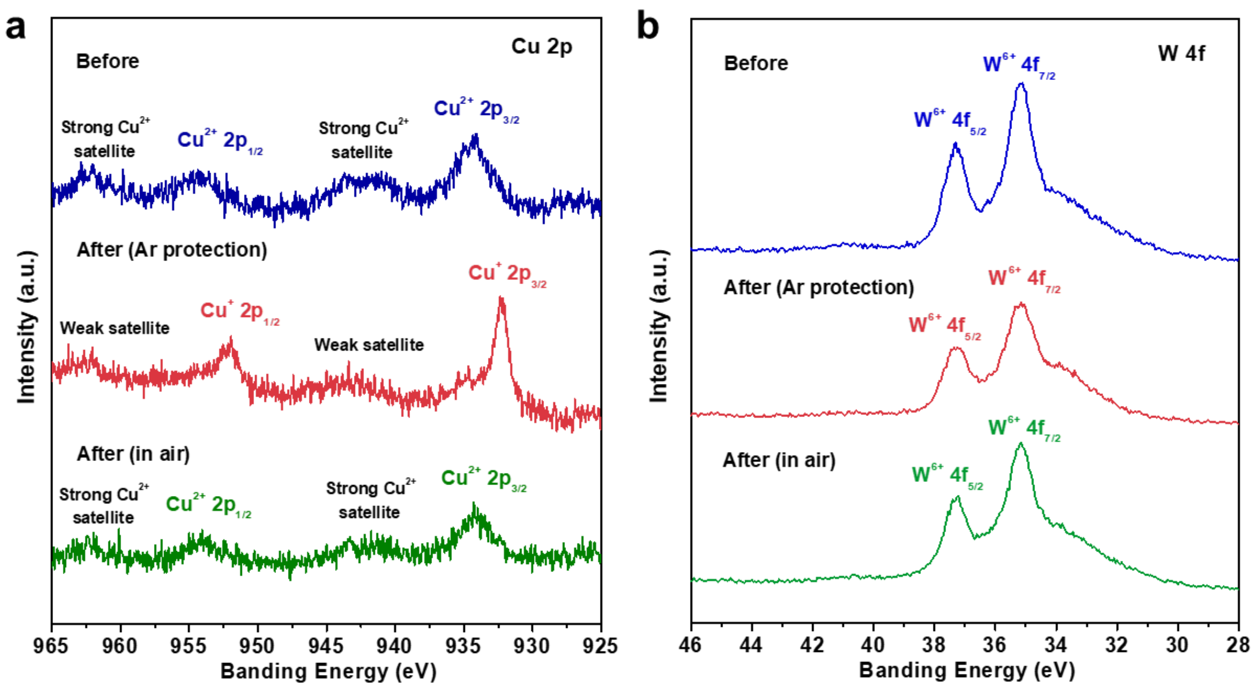

Supplementary Figure 39 XPS spectra of $CuWO_4$ before and after catalysis reaction (in Ar protection or in the air). XPS spectra of Cu 2p (a) and W 4f (b).

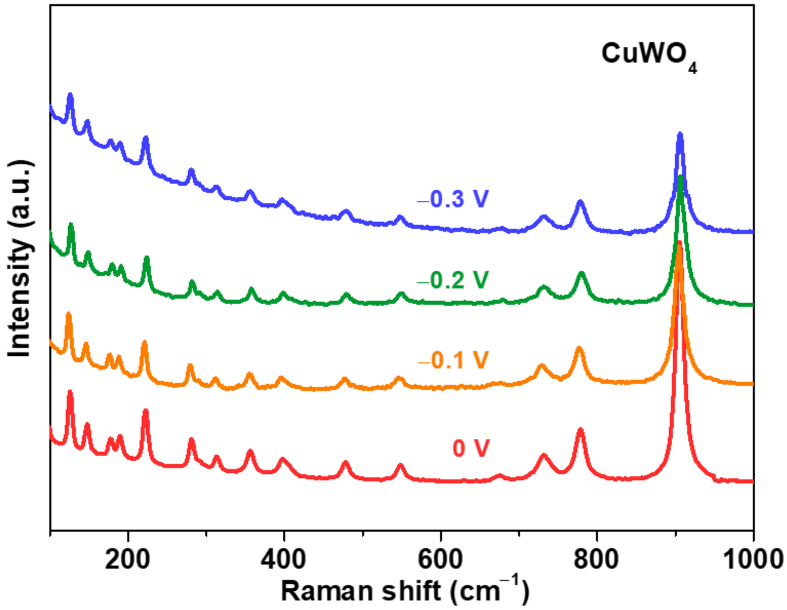

Supplementary Figure 40 In-situ Raman spectra of $CuWO_4$/CP electrodes in 0.1 M KNO_3 electrolyte with CO_2 feeding gas at different operating potentials.

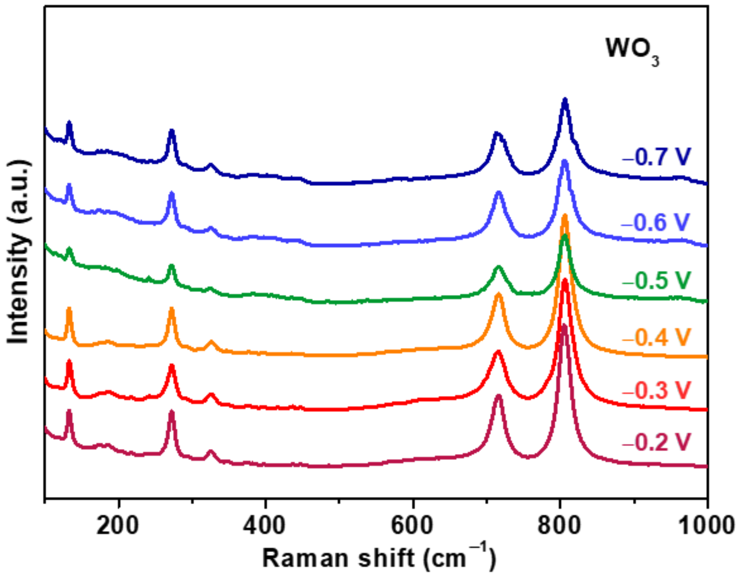

Supplementary Figure 41 In-situ Raman spectra of WO_3/CP electrodes in 0.1 M KNO_3 electrolyte with CO_2 feeding gas at different operating potentials.

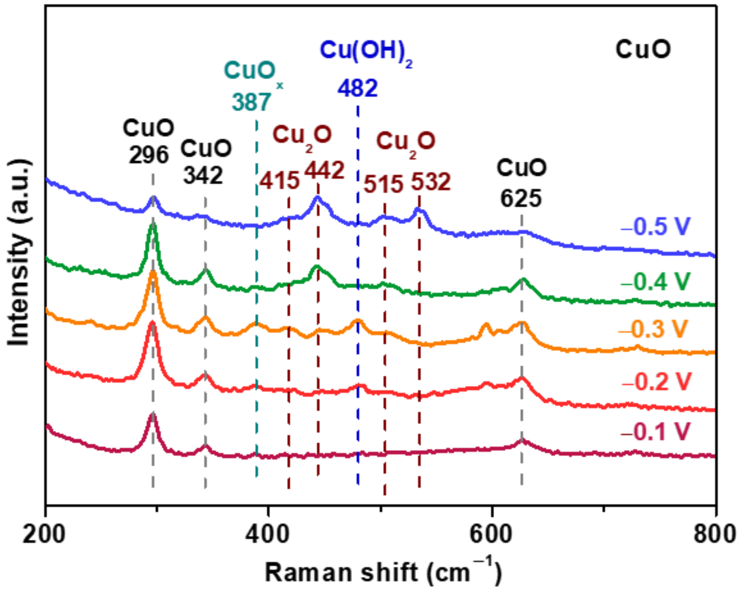

Supplementary Figure 42 In-situ Raman spectra of CuO/CP electrodes in 0.1 M KNO_3 electrolyte with CO_2 feeding gas at different operating potentials.

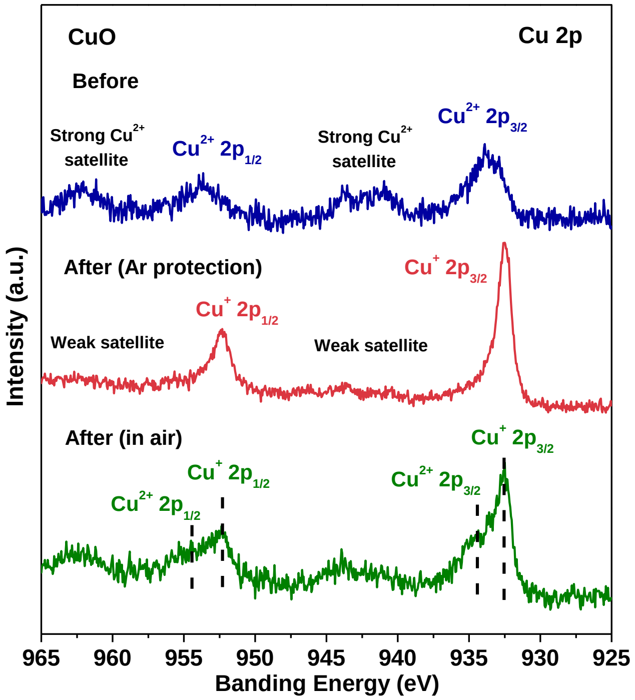

Supplementary Figure 43 Cu 2p XPS spectra of CuO before and after catalysis reaction (in Ar protection or in the air).

Supplementary Table 1 Electrocatalytic performance of coupling CO_2 and oxynitride to urea performance on heterogeneous catalytic systems in published works.

Catalyst	Electrolyte	Reactants	Operating potential (V vs. RHE)	FE (%)	Partial current density (mA cm^{-2})	Quantitative methods	Ref.
**$CuWO_4$**	0.1 M KNO_3	NO_3^-, CO_2	−0.2	70.9	0.95	Colorimetric method	This work
**$CuWO_4$**	0.1 M $K^{15}NO_3$	$^{15}NO_3^-$, CO_2	−0.2	70.1	0.95	NMR	
**$In(OH)_3$**	0.1 M KNO_3	NO_3^-, CO_2	−0.6	53	0.3	Colorimetric method	[1]
**V_O-InOOH**	0.1 M KNO_3	NO_3^-, CO_2	−0.5	51	0.9	Colorimetric method	[2]
**Cu-GS**	0.1 M $KHCO_3$ + 0.1 M KNO_3	NO_3^-, CO_2	−0.9	28	27	Colorimetric method	[3]
**Cu@Zn Nanowires**	0.2 M $KHCO_3$	NO_3^-, CO_2	−1.02	9.28	3.13	Liquid chromatography	[4]
**AuPd nanoalloy**	0.075 M $KHCO_3$ + 0.025 M KNO_3	NO_3^-, CO_2	−0.4	15.6	–	Colorimetric method	[5]
**Te-doped Pd**	0.1 M $KHCO_3$ + 0.01 M KNO_2	NO_2^-, CO_2	−1.1	12.2	–	Colorimetric method	[6]
**ZnO-V_O**	0.2 M $NaHCO_3$ + 0.1 M $NaNO_2$	NO_2^-, CO_2	−0.79	23.2	5.3	Liquid chromatography	[7]
**Zn**	0.2 M $KHCO_3$ + 0.02 M KNO_3	NO_3^-, CO_2	−1.3	35	–	Colorimetric method	[8]
**Ni-Pc**	0.2 M $KHCO_3$ + 0.02 M KNO_2	NO_2^-, CO_2	−1.1	41	–	Colorimetric method	[9]
**TiO_2/Nafion**	0.1 M KNO_3	NO_3^-, CO_2	−0.5	40	0.8	Colorimetric method	[10]
**TiO_2 nanotubes -V_O/Cu**	0.2 M $KHCO_3$ + 0.02 M KNO_2	NO_2^-, CO_2	−0.4	43.1	3.2	Colorimetric method	[11]
**Cd**	0.2 M $KHCO_3$ + 0.02 M KNO_3	NO_3^-, CO_2	−0.6	55	–	Colorimetric method	[12]
**AuCu SANFs**	0.5 M $KHCO_3$ + 0.02 M KNO_2	NO_2^-, CO_2	−1.0	24.7	20	Colorimetric method	[13]
**Zn foil**	0.2 M $KHCO_3$	NO, CO_2	−0.92	11.2	40	Liquid chromatography	[14]
**B-FeNi-DASC**	0.1 M $KHCO_3$ + 0.05 M KNO_3	NO_3^-, CO_2	−1.5	17.8	43	Colorimetric method	[15]
**Vo-CeO_2**	0.1 M $KHCO_3$ + 0.05 M KNO_3	NO_3^-, CO_2	-1.6	4.8	40	Colorimetric method	[16]

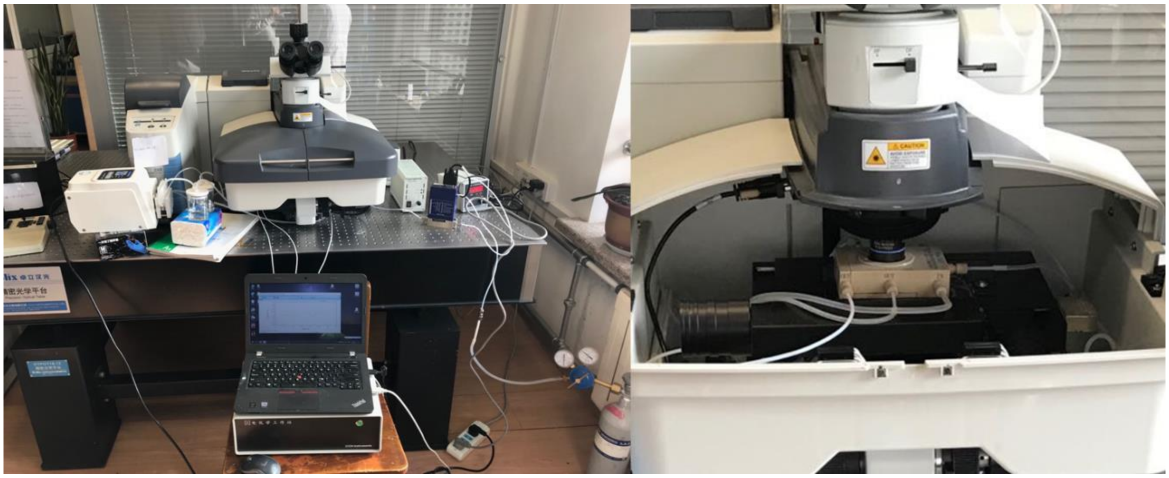

Supplementary Figure 44 Images of the experimental set-up and cell for operando Raman test.

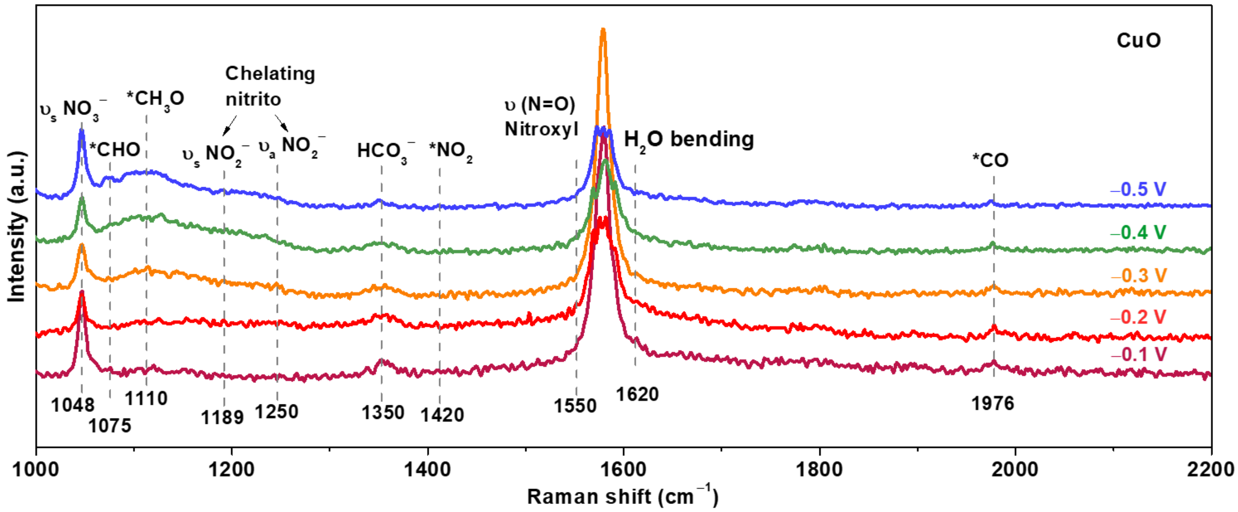

Supplementary Figure 45 Operando Raman spectra of CuO/CP electrodes in 0.1 M KNO_3 with CO_2 bubbling at different applied potentials.

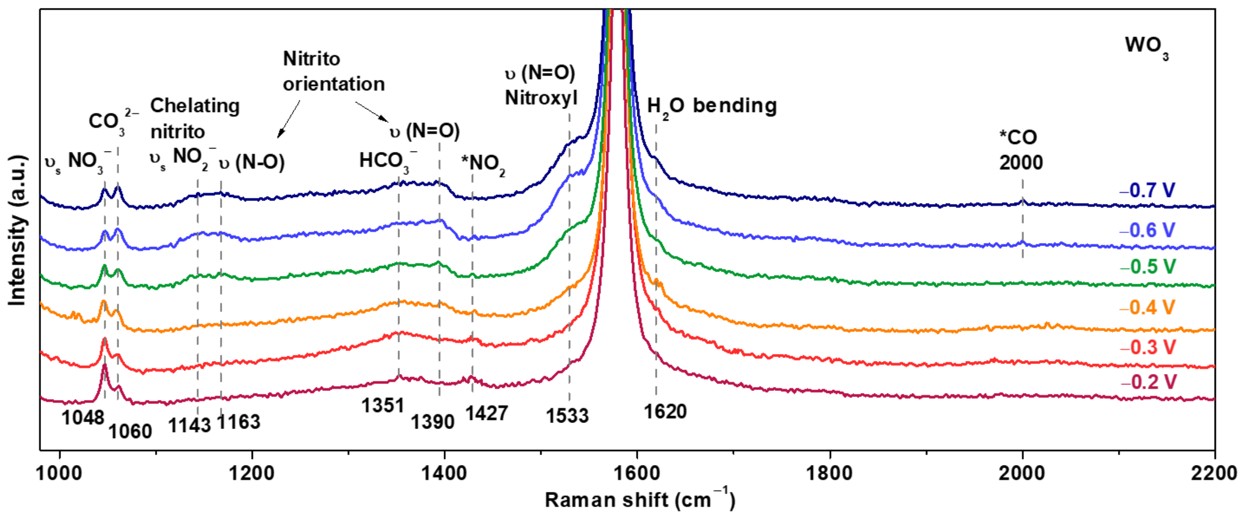

Supplementary Figure 46 Operando Raman spectra of WO_3/CP electrodes in 0.1 M KNO_3 with CO_2 bubbling at different applied potentials.

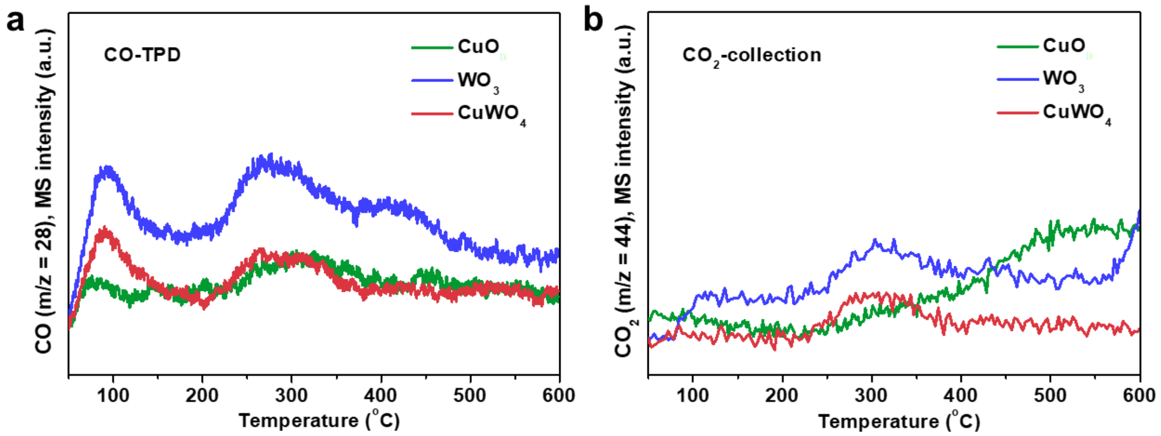

Supplementary Figure 47 The m/z signals of 28 (a) and 44 (b) in CO-temperature programmed desorption (TPD) of $CuWO_4$, CuO and WO_3.

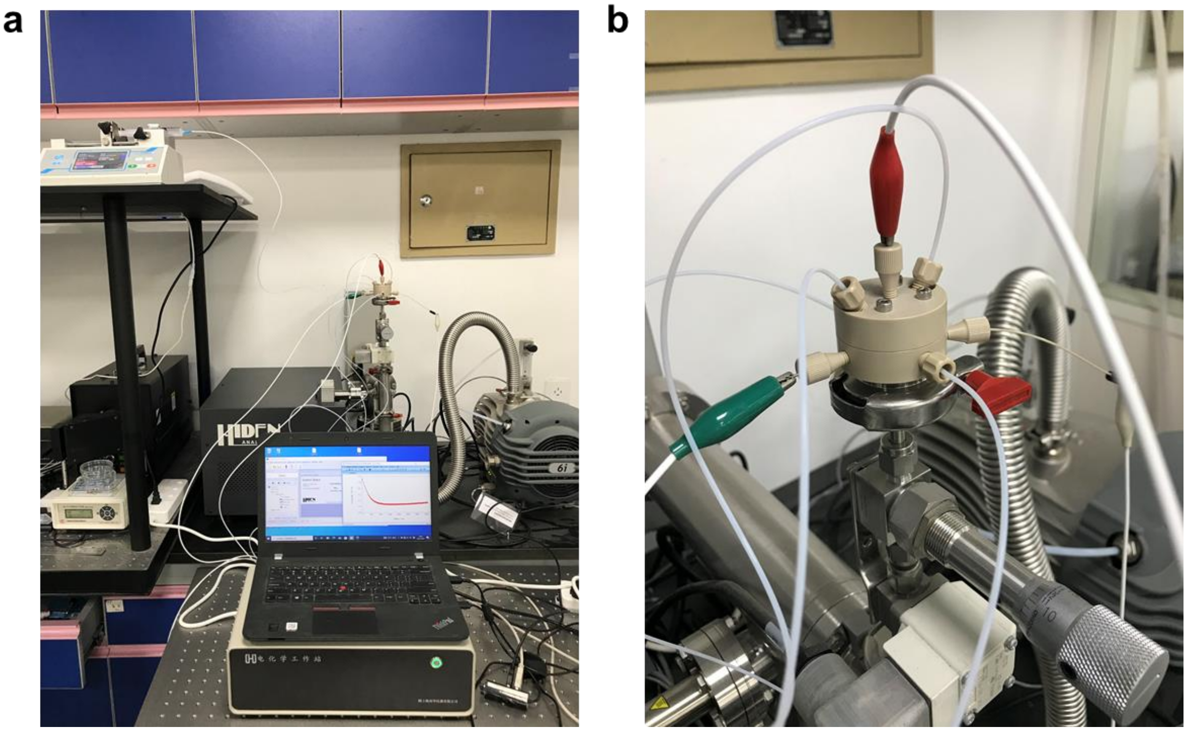

Supplementary Figure 48 Photograph of (a) differential electrochemical mass spectrometer and (b) DEMS cell.

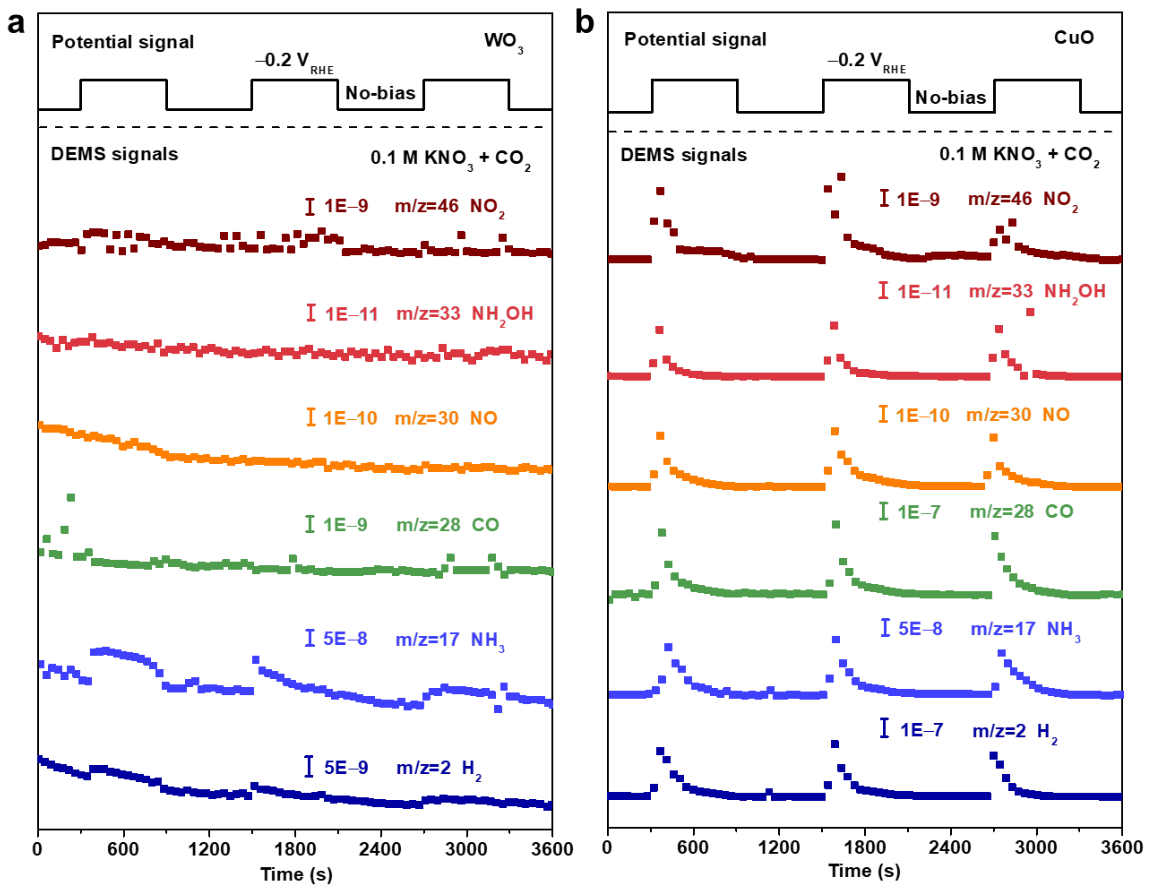

Supplementary Figure 49 Online DEMS of (a) WO_4 and (b) CuO in 0.1 M KNO_3 with saturated CO_2 at −0.2 V versus RHE.

Supplementary Table 2 The list of controlled experiments carried out to elucidate the mechanism of urea production.

Catalysts	C source	N source	V versus RHE	Urea?	Electrolyte
**$CuWO_4$**	CO_2	NO_3^-	−0.2	Yes	0.1 M KNO_3
	CO_3^-	NO_3^-	−0.2	Yes	0.05 M KCO_3 + 0.1 M KNO_3
	HCO_3^-	NO_3^-	−0.2	Yes	0.05 M $KHCO_3$ + 0.1 M KNO_3
	CO	NO_3^-	−0.2	Yes	0.1 M KNO_3
	HCHO	NO_3^-	0.0 to −0.4	NO	0.05 M HCHO + 0.1 M KNO_3
	CH_3OH	NO_3^-	0.0 to −0.4	NO	0.05 M CH_3OH + 0.1 M KNO_3
	CO_2	NO_2	−0.2	Yes	0.1 M $KHCO_3$
	CO_2	NO_2^-	0.0 to −0.4	NO	0.1 M KNO_2
	CO_2	NO	0.0 to −0.4	NO	0.1 M $KHCO_3$
	CO_2	NH_2OH	0.0 to −0.4	NO	0.05 M NH_2OH + 0.1 M $KHCO_3$ (adjust the pH to 7)
	CO_2	NH_3	0.0 to −0.4	NO	0.05 M NH_4Cl + 0.1 M $KHCO_3$ (adjust the pH to 7)
	CO	NO_2	−0.2	Yes	0.1 M phosphate buffered solution (pH=6.8)

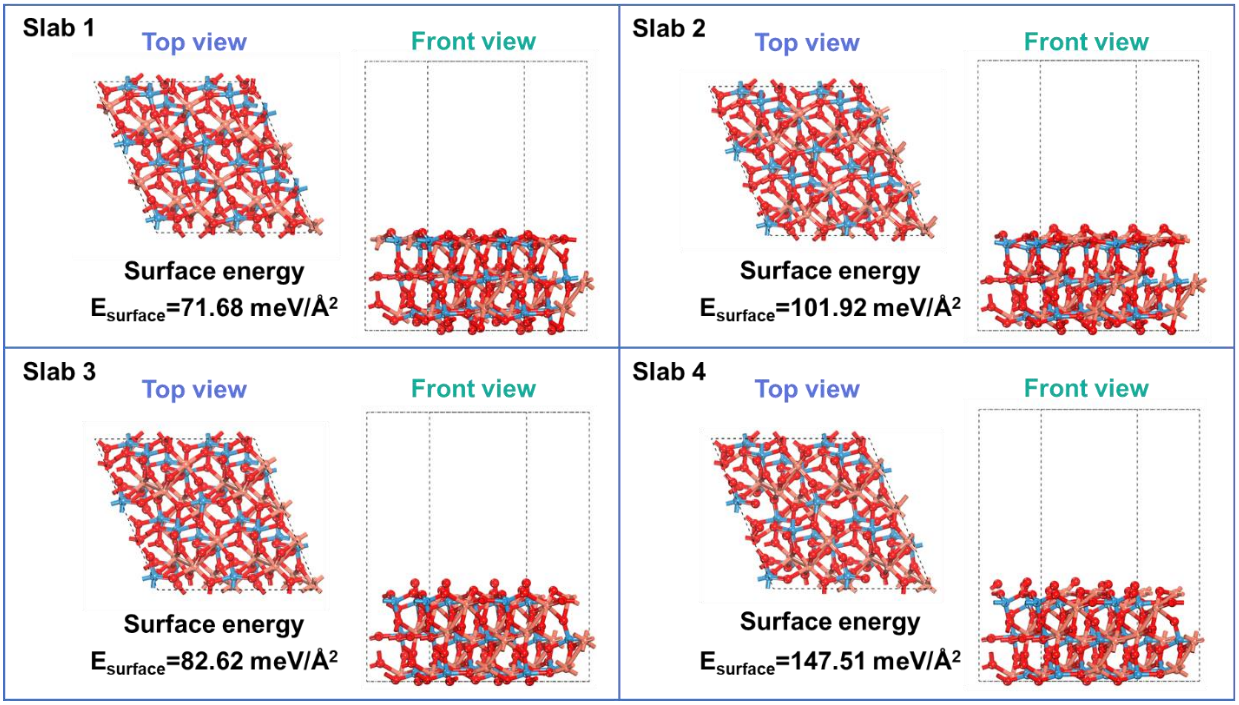

Supplementary Figure 50 The construction of four possible $CuWO_4$ (111) surfaces and their surface energy. Red, orange and cyan balls represent O, Cu and W, respectively.

Supplementary Discussion

Calculation of surface energy

For the four different $CuWO_4$ (111) surfaces, the surface energy (E_{surf}) is calculated as

$$E_{surf} = \frac{1}{2A}(E_{slab} - N * E_{bulk}) \quad (1)$$

where E_{slab} is the total energy of a surface periodic slab, E_{bulk} refers to the energy per atom of the bulk metal, N denotes the number of atoms in the surface slab, and A is the cross-sectional area of the surface slab unit cell.

As shown in Figure **Supplementary Fig. 50**, the **slab 1** has the lowest surface energy (71.68 meV/Å^2), implying its geometry structure to be the most stable one among those different $CuWO_4$ (111) surfaces.

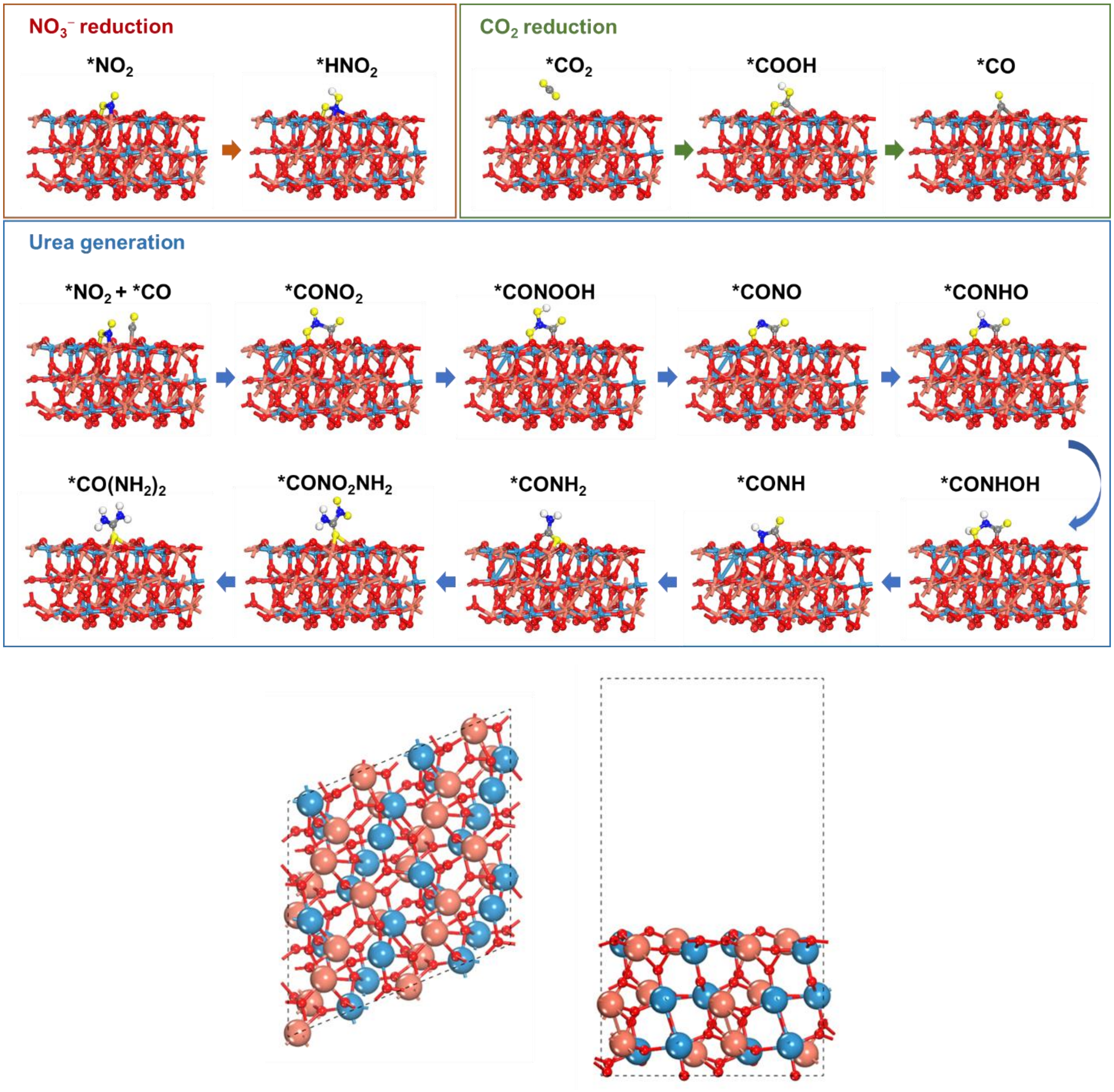

Supplementary Figure 51 The possible reaction mechanism, intermediate states with the lowest energy and an enlarged version of the $CuWO_4$ (111) facet. Gray, blue, red, orange, cyan, and yellow balls represent C, N, lattice O, Cu, W, and adsorbate O, respectively.

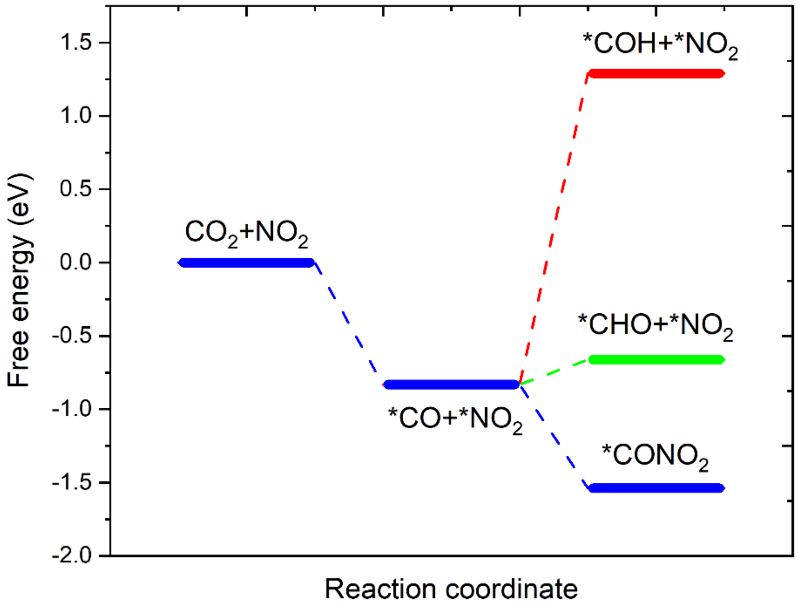

Supplementary Figure 52. Free-energy diagram for CO hydrogenation and C–N coupling process.

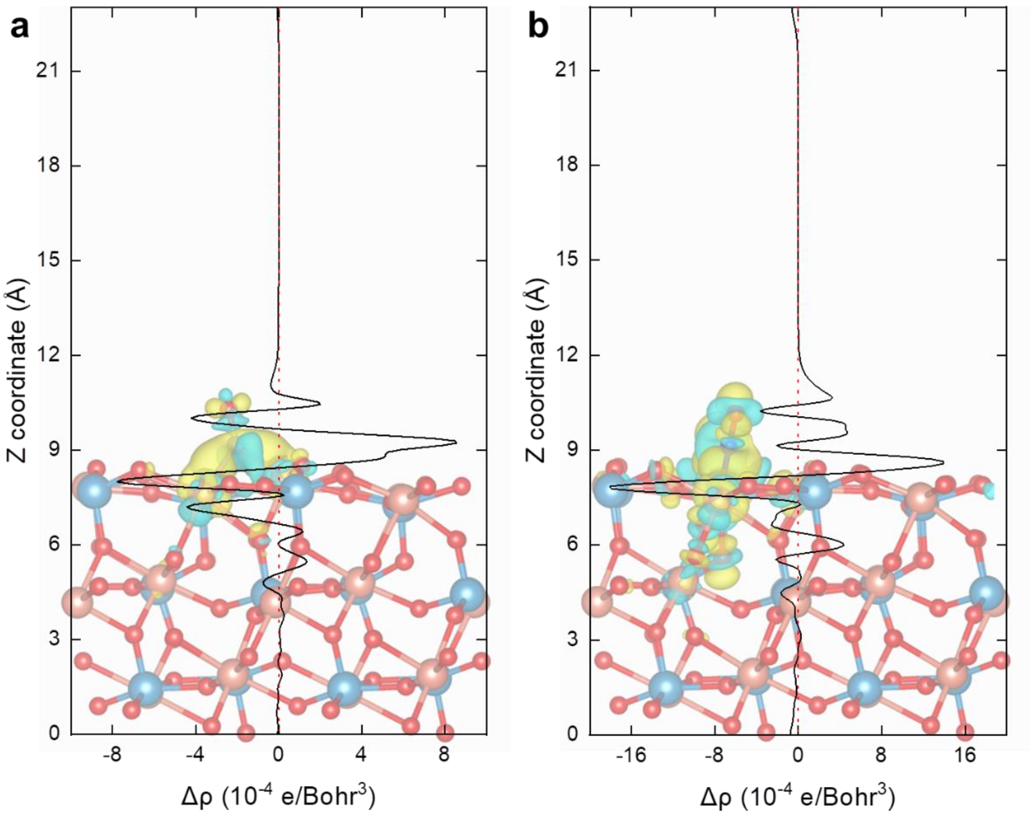

Supplementary Figure 53 The planar-averaged electron density difference Δρ and the charge density difference of (a) the *CO and (b) *NO_2 adsorbed on the $CuWO_4$ (111) surface. The iso-value is 0.002 e/$Å^3$. Cyan and yellow represent a depletion and an accumulation of electrons, respectively.

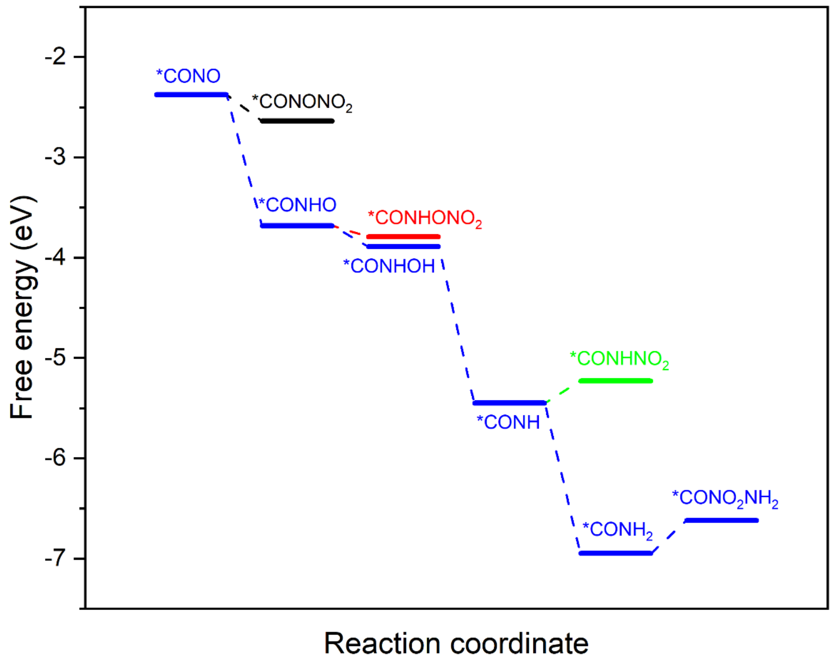

Supplementary Figure 54. Free-energy diagram for the second C–N bond formed.

Supplementary References

1. C. Lv, L. Zhong, H. Liu, Z. Fang, C. Yan, M. Chen, Y. Kong, C. Lee, D. Liu, S. Li, J. Liu, L. Song, G. Chen, Q. Yan and G. Yu, *Nat. Sustain.* **2021**, *4*, 868-876.
2. C. Lv, C. Lee, L. Zhong, H. Liu, J. Liu, L. Yang, C. Yan, W. Yu, H. H. Hng, Z. Qi, L. Song, S. Li, K. P. Loh, Q. Yan and G. Yu, *ACS Nano* **2022**, *16*, 8213-8222.
3. J. Leverett, T. Tran-Phu, J. A. Yuwono, P. Kumar, C. Kim, Q. Zhai, C. Han, J. Qu, J. Cairney, A. N. Simonov, R. K. Hocking, L. Dai, R. Daiyan and R. Amal, *Adv. Energy Mater.* **2022**, *12*, 2201500.
4. N. Meng, X. Ma, C. Wang, Y. Wang, R. Yang, J. Shao, Y. Huang, Y. Xu, B. Zhang and Y. Yu, *ACS Nano*, **2022**, *16*, 9095-9104.
5. H. Wang, Y. Jiang, S. Li, F. Gou, X. Liu, Y. Jiang, W. Luo, W. Shen, R. He and M. Li, *Appl. Catal. B: Environ.* **2022**, *318*, 121819.
6. Y. Feng, H. Yang, Y. Zhang, X. Huang, L. Li, T. Cheng and Q. Shao, *Nano Lett.* **2020**, *20*, 8282-8289.
7. N. Meng, Y. Huang, Y. Liu, Y. Yu and B. Zhang, *Cell Rep. Phys. Sci.* **2021**, *2*, 100378.
8. M. Shibata, K. Yoshida and N. Furuya, *J. Electrochem. Soc.* **1998**, *145*, 2348-2353.
9. M. Shibata and N. Furuya, *J. Electroanal. Chem.* **2001**, *507*, 177-184.
10. D. Saravanakumar, J. Song, S. Lee, N. H. Hur and W. Shin, *ChemSusChem* **2017**, *10*, 3999-4003.
11. N. Cao, Y. Quan, A. Guan, C. Yang, Y. Ji, L. Zhang and G. Zheng, *J. Colloid Interf. Sci.* **2020**, *577*, 109-114.
12. M. Shibata, K. Yoshida and N. Furuya, *J. Electrochem. Soc.* **1998**, *145*, 595-600.
13. S. Liu, S. Yin, Z. Wang, Y. Xu, X. Li, L. Wang and H. Wang, *Cell Rep. Phys. Sci.* **2022**, *3*, 100869.
14. Y. Huang, R. Yang, C. Wang, N. Meng, Y. Shi, Y. Yu and B. Zhang, *ACS Energy Lett.* **2022**, *7*, 284-291.
15. X. Zhang, X. Zhu, S. Bo, C. Chen, M. Qiu, X. Wei, N. He, C. Xie, W. Chen, J. Zheng, P. Chen, S. P. Jiang, Y. Li, Q. Liu and S. Wang, *Nat. Commun.* **2022**, *13*, 5337.
16. X. Wei, X. Wen, Y. Liu, C. Chen, C. Xie, D. Wang, M. Qiu, N. He, P. Zhou, W. Chen, J. Cheng, H. Lin, J. Jia, X.-Z. Fu, S. Wang, *J. Am. Chem. Soc.* **2022**, *144*, 11530-11535.
